# Supplementary material for: Cultural stigma, psychological distress and help-seeking: Moderating role of self-esteem and self-stigma in Lebanon
Source: PLoS One. 2025 Sep 25;20(9):e0315063. doi: 10.1371/journal.pone.0315063 (PMC12463265; doi:10.1371/journal.pone.0315063)
Supplement: S2 File — Linear regression with help-seeking attitude (dependent variable), psychological distress (independent variable), and self-esteem (moderator).Supplementary table S2 Table 2 Linear regression with help-seeking attitude (dependent variable), psychological distress (independent variable), and self-stigma (moderator). (DOCX) [file pone.0315063.s002.docx]

| **Table 1. Linear regressions taking help-seeking attitude as the dependent variable, psychological distress as the independent variable and self-esteem as the moderator.** | | | | |
| --- | --- | --- | --- | --- |
| **Variable** | **Unstandardized Beta** | **Standardized Beta** | ***p*** | **95% CI** |
| **Step 1: Results of the baseline model with only control variables (Nagelkerke R^2^ = .181)** | | | | |
| Age (20-29 vs 18-19 years) | -.48 | -.20 | .009 | -.84; -.12 |
| Age (30-39 vs 18-19 years) | .10 | .02 | .763 | -.54; .73 |
| Age (40 vs 18-19 years) | .79 | .10 | .179 | -.37; 1.96 |
| Sex (females vs males*) | .14 | .10 | .128 | -.04; .33 |
| Education (bachelor vs high school*) | -.83 | -.49 | **<.001** | -1.22; -.45 |
| Education (master vs high school*) | -.55 | -.29 | **.012** | -.97; -.12 |
| Education (PhD vs high school*) | -.44 | -.05 | .474 | -1.64; .76 |
| **Step 2: Results of the model with main effects included (Nagelkerke R^2^ = .397)** | | | | |
| Age (20-29 vs 18-19 years) | -.24 | -.10 | .135 | -.56; .08 |
| Age (30-39 vs 18-19 years) | .39 | .09 | .166 | -.16; .94 |
| Age (40 vs 18-19 years) | 1.41 | .17 | **.007** | .40; 2.42 |
| Sex (females vs males*) | -.03 | -.02 | .698 | -.20; .13 |
| Education (bachelor vs high school*) | -.60 | -.35 | **<.001** | -.93; -.26 |
| Education (master vs high school*) | -.43 | -.23 | **.024** | -.80; -.06 |
| Education (PhD vs high school*) | -.134 | -.16 | **.013** | -2.39; -.29 |
| Psychological distress | -.21 | -.27 | **<.001** | -.30; -.12 |
| Self-esteem | .11 | .36 | **<.001** | .08; .15 |
| **Step 3: Results of the model with interaction terms added (Nagelkerke R^2^ = .437)** | | | | |
| Age (20-29 vs 18-19 years) | -.20 | -.08 | .205 | -.50; .11 |
| Age (30-39 vs 18-19 years) | .48 | .11 | .077 | -.05; 1.02 |
| Age (40 vs 18-19 years) | 1.67 | .20 | **<.001** | .69; 2.66 |
| Sex (females vs males*) | -.06 | -.04 | .482 | -.22; .10 |
| Education (bachelor vs high school*) | -.64 | -.38 | **<.001** | -.96; -.32 |
| Education (master vs high school*) | -.61 | -.32 | **.001** | -.99; -.24 |
| Education (PhD vs high school*) | -1.15 | -.14 | **.028** | -2.17; -.12 |
| Psychological distress | -2.19 | -2.75 | **<.001** | -3.15; -1.23 |
| Self-esteem | -.11 | -.36 | .054 | -.23; .002 |
| Interaction psychological distress by self-esteem | .08 | 2.40 | **<.001** | .04; .12 |

Numbers in bold indicate significant *p* values.

| **Table 2. Linear regressions taking help-seeking attitude as the dependent variable, psychological distress as the independent variable and self-stigma as the moderator.** | | | | |
| --- | --- | --- | --- | --- |
| **Variable** | **Unstandardized Beta** | **Standardized Beta** | ***p*** | **95% CI** |
| **Step 1: Results of the baseline model with only control variables (Nagelkerke R^2^ = .181)** | | | | |
| Age (20-29 vs 18-19 years) | -.48 | -.20 | **.009** | -.84; -.12 |
| Age (30-39 vs 18-19 years) | .10 | .02 | .763 | -.54; .73 |
| Age (40 vs 18-19 years) | .79 | .10 | .179 | -.37; 1.96 |
| Sex (females vs males*) | .14 | .10 | .128 | -.04; .33 |
| Education (bachelor vs high school*) | -.83 | -.49 | **<.001** | -1.22; -.45 |
| Education (master vs high school*) | -.55 | -.29 | **.012** | -.97; -.12 |
| Education (PhD vs high school*) | -.44 | -.05 | .474 | -1.64; .76 |
| **Step 2: Results of the model with main effects included (Nagelkerke R^2^ = .340)** | | | | |
| Age (20-29 vs 18-19 years) | -.24 | -.10 | .147 | -.57; .09 |
| Age (30-39 vs 18-19 years) | .35 | .08 | .231 | -.23; .93 |
| Age (40 vs 18-19 years) | 1.21 | .15 | **.024** | .16; 2.27 |
| Sex (females vs males*) | .07 | .05 | .427 | -.10; .24 |
| Education (bachelor vs high school*) | -.54 | -.32 | **.003** | -.90; -.18 |
| Education (master vs high school*) | -.20 | -.10 | .329 | -.59; .20 |
| Education (PhD vs high school*) | -1.01 | -.12 | .070 | -2.10; .09 |
| Psychological distress | -.13 | -.16 | .051 | -.25; .001 |
| Self-stigma | .14 | .31 | **<.001** | .07; .21 |
| **Step 3: Results of the model with interaction terms added (Nagelkerke R^2^ = .360)** | | | | |
| Age (20-29 vs 18-19 years) | -.20 | -.08 | .238 | -.53; .13 |
| Age (30-39 vs 18-19 years) | .40 | .09 | .164 | -.17; .98 |
| Age (40 vs 18-19 years) | 1.42 | .17 | **.008** | .37; 2.47 |
| Sex (females vs males*) | .06 | .04 | .501 | -.11; .22 |
| Education (bachelor vs high school*) | -.50 | -.29 | **.007** | -.85; -.14 |
| Education (master vs high school*) | -.14 | -.07 | .485 | -.53; .25 |
| Education (PhD vs high school*) | -.86 | -.10 | .121 | -1.94; .23 |
| Psychological distress | -.37 | -.46 | **<.001** | -.58; -.15 |
| Self-stigma | -.03 | -.07 | .658 | -.18; .11 |
| Interaction psychological distress by self-stigma | .07 | .31 | **.008** | .02; .12 |

Numbers in bold indicate significant *p* values.
