# Supplementary material for: Cultural stigma, psychological distress and help-seeking: Moderating role of self-esteem and self-stigma in Lebanon
Source: PLoS One. 2025 Sep 25;20(9):e0315063. doi: 10.1371/journal.pone.0315063 (PMC12463265; doi:10.1371/journal.pone.0315063)
Supplement: Attachment — Submitted filename: Peer Review Report.pdf [file pone.0315063.s003.pdf]

Peer Review Comments

Summary of the research

Main Issues, Arguments, and Conclusions

This study explores the connections between Cultural Stigma, Psychological Distress, and Help-Seeking Behavior, with a particular focus on the moderating roles of Self-esteem and Self-stigma. Using a cross-sectional online survey of 245 young Lebanese adults, the study found significant negative associations between cultural stigma and both psychological distress and help-seeking attitudes. Moreover, self-esteem and self-stigma affected these relationships, increasing psychological distress and reducing help-seeking intentions when cultural stigma was high and self-esteem was low or self-stigma was high.

The findings highlight that the harmful impact of cultural stigma on mental health is particularly strong in the Lebanese cultural context. The moderating roles of self-esteem and self-stigma provide important insights for designing culturally relevant mental health interventions. The study suggests that mental health interventions should consider cultural differences, focusing on improving self-esteem and managing self-stigma to help individuals cope with stigma-related distress and increase their willingness to seek help.

Positioning and Contributions in the Existing Literature

This study adds value to the existing literature by examining the dynamics of cultural stigma, psychological distress, and help-seeking behavior within the specific cultural context of Lebanon, a non-Western setting that has been underrepresented in mental health stigma research. Unlike much of the existing literature based in Western contexts, this study makes the following key contributions. First, it broadens the cross-cultural perspective on mental health stigma by examining how cultural factors shape stigma and its consequences in Lebanon's social and cultural environment. The empirical data from this non-Western context enhance cross-cultural comparisons in this field.

Second, the study improves our understanding of how self-esteem and self-stigma influence the effects of cultural stigma on psychological distress and help-seeking behavior. This theoretical insight supports the use of moderation effects when designing targeted mental health interventions.

Finally, the study provides practical suggestions for tackling cultural stigma, particularly for groups with high levels of stigma. By emphasizing the importance of strengthening self-esteem and reducing self-stigma, the study offers useful guidance for policymakers and mental health professionals who aim to design culturally appropriate interventions.

Strengths and Weaknesses

This study has several strengths. It addresses an important issue by exploring how cultural stigma affects mental health and help-seeking behaviors, with a focus on the moderating roles of self-esteem and self-stigma. This focus enhances our understanding of the psychological processes involved in mental health and offers valuable insights for future research and interventions. The study also uses well-established psychological measurement tools, such as the Rosenberg Self-esteem Scale and the Kessler Psychological Distress Scale, to ensure the accuracy and reliability of the data. Furthermore, the study contributes to the broader field of cross-cultural research by exploring cultural stigma in a non-Western context, specifically Lebanon, which fills a gap in the literature.

However, there are areas for improvement. One limitation is the representativeness of the sample. The study primarily focuses on young adults aged 20-29, which may limit the ability to apply the findings to other age groups or social demographics. Additionally, the reliance on online recruitment via social media platforms may introduce bias, narrowing the diversity of the sample.

Another limitation lies in the theoretical analysis of the moderating mechanisms. While the study identifies the roles of self-esteem and self-stigma as moderators, it does not explore these effects in enough detail. This lack of explanation means that the reader does not fully understand why or how these moderators affect the relationships between cultural stigma and mental health outcomes.

Finally, the study could go deeper into the specific cultural factors in Lebanon, such as family values and social norms, that influence the development and persistence of cultural stigma. A more detailed exploration of these cultural factors could provide richer insights into the broader implications of the findings.

General Recommendations

To improve the quality and impact of the research, several suggestions are made. First, expanding the sample to include a wider range of participants, such as those from different age groups and professions, would improve the applicability of the findings.

Second, a more thorough theoretical analysis of the moderating mechanisms would strengthen the study's conceptual foundation. By drawing on established theories, such as stigma management theory or social cognitive theory, the study could better explain the internal logic behind the moderating effects observed.

Third, incorporating a detailed examination of Lebanese cultural norms and their role in shaping stigma would enhance the cultural analysis and strengthen the study's cross-cultural contributions. Fourth, the study could benefit from more specific and actionable recommendations for interventions. These could include educational programs aimed at improving self-esteem, reducing stigma, and raising mental health awareness through community campaigns.

Fourth, increasing transparency in the methodology, particularly regarding participant recruitment and data collection processes, would improve the study's credibility and replicability.

Lastly, improving the clarity and consistency of the language throughout the paper is essential. The English language expression, including punctuation, grammar, and overall writing style, should adhere more closely to academic norms for journal publications.

In summary, this study offers valuable insights into the dynamics of cultural stigma, self-esteem, and self-stigma in a non-Western cultural context. While it successfully identifies key relationships and provides practical recommendations, addressing the limitations in sample representativeness, theoretical depth, and cultural context would

significantly enhance its contribution to the field. The study holds great potential to inform culturally sensitive mental health interventions and inspire further research in cross-cultural psychology.

Examples and evidence

Literature Review Section

Major Issues

(1)The literature review section is not grounded in a clear theoretical framework, resulting in insufficient theoretical depth throughout the review. It is recommended that the author use relevant theories to strengthen the relationship statements between the variables of interest, thus laying a more solid theoretical foundation for proposing the hypotheses of this study.

(2) Lack of specificity in describing the research gap. Although the manuscript points out that "research on the influence of stigma on mental health and help-seeking behavior is scarce" in Lebanon, it fails to specify the limitations of existing studies (e.g., Wehbe, 2011; Hassan, 2015), which affects the positioning and value of the current research.

(3) Overly broad statement of research objectives. The manuscript mentions at the end that "The purpose of this research is thus to provide empirical data on the influence of stigma on self-esteem and help-seeking behaviors," but it does not propose specific research questions or hypotheses.

(4) Absence of research hypotheses: The literature review section does not explicitly state research hypotheses, although they can be inferred from the title and research content (e.g., the moderating roles of cultural stigma, self-esteem, and self-stigma). The lack of clear research hypotheses may result in a lack of theoretical support and explicit hypothesis testing in the results section, potentially leading to a more descriptive rather than hypothesis-driven study.

Minor Issues

(1) Regarding structure, the transition from global issues to the specific situation in

Lebanon is not smooth enough. It is suggested to use headings to structure this section and enhance its logical flow.

(2) Regarding data, the WHO (2019) data needs to be updated, and the data stating that "75-80% of people with mental disorders not receiving therapy" also requires a more recent source.

(3) Regarding local studies, the discussion of the Lebanese situation mainly relies on research by Karam et al. (2019) and Hassan (2015), and more recent local studies need to be added.

(4) Regarding writing expression, the use of the tense "has been recently investigated" does not match the cited 2009 study (Quinn & Chaudoir). From a cross-cultural perspective, the discussion of "global South and Arab countries" lacks a systematic comparison with other cultural contexts.

(5) Reliance on a single source for defining core concepts. The definition of social stigma in the manuscript only cites Watson et al. (2003): "social stigma refers to the public beliefs and impressions regarding a specific person or community," lacking support and in-depth discussion from more recent literature.

(6) Updating references: The overall literature in the review section needs to be supplemented with the latest literature relevant to the research topic.

Method Section

Major Issues

(1) The data collection period "between March 2023 and August 2024" is excessively long, and the actual data collection time frame needs to be clarified. An unusually long data collection period may affect the temporal consistency of the data, introduce potential time-effect biases, and indicate potential issues with the study execution process.

(2) The manuscript mentions, "Participants responded to the survey link shared via social media platforms such as Instagram and WhatsApp." The specific processes for participant recruitment and questionnaire distribution need to be clearly described: Were the questionnaires sent to pre-identified eligible participants via social media, or

was recruitment conducted openly on social media? This affects readers' understanding of the research methods and their judgment of the reliability of the results. The current content only lists the participant inclusion criteria and the number of excluded questionnaires, completely lacking a substantive description of the study execution process. This section should clearly state "how the study was conducted." It is recommended to further clarify how the research team reached potential participants, how participants were recruited, how the questionnaires were distributed, how data were collected, and what the entire research process was like. The absence of this key information makes it impossible to assess the repeatability and methodological quality of the study. The authors are advised to provide a complete description of the study execution process, rather than merely listing the inclusion criteria and exclusion numbers.

(3) There are several apparent issues in the "procedure" section of the manuscript. First, although the manuscript states, "Only participants who were above 18, living in Lebanon and diagnosed with at least one mental illness were eligible to participate," it does not further specify the diagnostic categories or present the distribution of specific mental disorder diagnoses among the participants. This lack of detailed diagnostic information may affect the interpretation of the study results. Second, there is ambiguity in the exclusion of invalid data in the data analysis: Although the article mentions that 14 participants were excluded due to incomplete questionnaires, the Study Design and Sample section still mentions a sample of 245 participants. This statement may cause ambiguity, and readers are unclear whether the final number of participants used for data analysis is 245 or 231 (the number after excluding invalid data). If the data analysis still used 245 participants (including invalid data), it would affect the accuracy of the results, as invalid data may lead to bias and affect the reliability of the conclusions.

Minor Issues

(1) There is an important lack of clarity in the description of the Self-Stigma Questionnaire (SSQ). In the current description, there is confusion regarding the

source of the scale and the purpose of the literature citation. Specifically, the text describes this 14-item scale and cites Ochoa et al. (2015) at the end, but fails to clearly distinguish the following key information: Who developed this scale? If it was developed by Ochoa et al., it should be explicitly stated when introducing the scale; if not, the original developers should be mentioned. What exactly is the study by Ochoa et al. (2015): the original scale development literature, or merely used to support the argument that "the scale has good reliability and validity across different populations and languages (Cronbach's alpha ranging from 0.75 to 0.901)"? This ambiguous citation style makes it difficult for readers to trace the original source of the scale and determine the specific role of the cited literature. The authors are advised to revise this content to clearly distinguish between the development source of the scale and subsequent validation studies, which is a basic requirement for the description of measurement tools in the methodology section.

(2) The manuscript mentions, "The attitude help seeking score was considered normally distributed since the skewness (= .001) and kurtosis (= -.702) values varied between -1 and +1 [ref]." The authors use skewness and kurtosis values within ±1 as the criterion for judging normal distribution but do not provide a reference to support this judgment criterion. The meaning of [ref] here seems to be not a formatting issue, but a substantive reference that needs to be supplemented.

(3) There are some formatting issues, such as in the sentence "It has been confirmed to be reliable and effective across a range of populations and languages. high level of internal consistency, and Cronbach's alpha coefficients ranging from 0.75 to 0.901 (Ochoa et al., 2015)," where the punctuation is incorrect.

Result Section

Major Issues:

(1) It is recommended that the textual description in the results section be expanded. Currently, the narrative is rather brief and overly reliant on tables. The following improvements could strengthen the section:

- Sample Characteristics:The current description simply states, "Two hundred forty-five young adults with diagnosed mental disorders participated in this study." It would be beneficial to provide a more comprehensive description of the sample characteristics.

- Bivariate Analysis Results:The existing statement, "The results showed that older participants and those with higher education compared to the other categories had significantly higher help-seeking attitude," is overly succinct. It is suggested to include specific information about the magnitude and patterns of these differences.

- Moderation Analysis Results: The current description, such as "The interaction self-esteem by psychological distress was significantly associated with help-seeking attitude (Beta = .08; t = 4.07; p < .001)," leans towards a technical tone. It is recommended to add an interpretation of the substantive significance of these results.

(2) The study identifies two significant moderation effects, and it is recommended that these results be presented visually for clarity. Currently, Table 4 presents the statistical results of the interaction effects (e.g., the interaction between self-esteem and psychological distress: Beta = .08; t = 4.07; p < .001), and Table 5 presents the conditional effects at different levels. To improve the presentation of these findings, it is suggested to create simple slope plots for both moderation models: Model 1: Plot for the moderation effect of self-esteem/ Model 2:Plot for the moderation effect of self-stigma.

(3) It is recommended that the authors include a path diagram in the results section to visualize the moderation effect model. The diagram could effectively demonstrate how the two independent variables (Cultural Stigma and Psychological Distress) influence the dependent variable (Help-Seeking Behavior) through the moderating variables (Self-esteem and Self-stigma). Adding such a visual representation in the results presentation would enhance the clarity of the findings, particularly in illustrating the complexity of multivariable interaction models, and help readers better understand the study's core results.

Minor Issues:

(1)To further enhance the informative value of the moderation analysis, it is

recommended to include additional model fit information. While the current analysis already presents solid evidence for the presence of moderation effects, including regression coefficients (Beta), significance tests (t-values and p-values), and 95% confidence intervals, additional details could be helpful for a more comprehensive understanding. The following supplementary information is suggested:

- Overall Model Fit Indicators: R^2 values at each step, the change in R^2 (ΔR^2) after adding the moderation term, and the corresponding F-change values with their significance levels.
- Stepwise Regression Information:
  - Step 1:Results of the baseline model with only control variables
  - Step 2: Results of the model with main effects included
  - Step 3: Results of the model with interaction terms added

(2) It is recommended that the table footnotes clearly display all three levels of statistical significance. Although there may not be any instances of *$p < .05$ in the results, it is standard practice in statistical reporting to include all levels of significance: *$p < .05$, **$p < .01$, ***$p < .001$. Therefore, the table footnotes should be revised to include these complete descriptions of statistical significance.

Discussion Section

Major Issues

(1)The discussion section lacks a clear articulation of the theoretical framework underpinning this study. It is suggested that the authors dedicate a section at the beginning of the discussion to outline the core theoretical framework of the study, clarifying the interrelations of different theories, in order to construct a complete theoretical analysis framework.

Specifically, the discussion of the relationships between stigma, self-esteem, self-stigma, and help-seeking intentions is not closely linked to specific theoretical perspectives. The current discussion primarily relies on previous empirical research findings to support the viewpoints, but it lacks a deep analysis of the theoretical logic behind these results. As a result, readers may find it difficult to understand the correspondence and connection between the study's findings and the key theories. It is

recommended that the discussion of key findings be tightly integrated with specific theoretical perspectives, using theoretical concepts and propositions to interpret the empirical results, thereby highlighting the alignment between the research findings and theory.

(2)The discussion currently focuses mainly on the implications of the research findings for understanding the relationships between stigma, self-esteem, self-stigma, psychological distress, and help-seeking intentions, as well as how the study adds to and extends existing research. While these discussions are important, the practical application of the research knowledge is not sufficiently addressed. Given that educational level is an important factor influencing help-seeking attitudes, the authors should provide more targeted and actionable recommendations for improving educational practices based on the study's results. At present, this aspect is relatively vague and underdeveloped. For example, the authors mention that "education on mental health literacy and service availability is also needed to increase help-seeking behavior," but do not provide specific suggestions on how to carry out targeted educational interventions.

Given that young people are typically in higher education or vocational training, it is particularly necessary and urgent to discuss how the findings could be applied to mental health curricula in schools, the provision of mental health services, and student counseling services. If the authors could offer practical and feasible recommendations for educators and policymakers, this would significantly enhance the practical value of the research.

Additionally, although the authors mention the cultural context of the study (Lebanon) in the case and background introduction, this context is not sufficiently explored in the discussion of practical implications. Educational interventions addressing mental illness stigma and help-seeking in different cultural settings may require distinct strategies and approaches. If the authors consider the cultural characteristics of Lebanon and offer constructive suggestions on how to adapt educational practices accordingly, the practical guidance of the paper would be further strengthened.

Minor Issues

(1)The logical organization and transitions between different points in the discussion need to be improved. There is a sense that the sections are somewhat disjointed, lacking necessary transitions and connections. It is recommended that the authors adjust the structure of the text to enhance the logical flow between the main findings. One approach could be to first summarize the key results and then provide a detailed discussion of each. Additionally, transitional sentences should be added between different sections to tightly link the content.

(2) The discussion of limitations and future research directions needs to be further clarified. The transition between the two sections should be improved. In addition to pointing out the study's limitations, the authors should analyze how these limitations might affect the interpretation of the results, and use these analyses as a basis for suggesting future research directions. For example, while the sample of 245 participants is adequate for data analysis, it may be too small for studies involving multiple variables and moderation effects. The authors should acknowledge the limitation of the small sample size and explain how it may influence the study's results.

References Section

Major Issue: The references section contains several outdated citations that require updating. Notably, core references such as Link & Phelan (2001) and Wright et al. (2000) are cited, which may no longer reflect the most recent developments in the field. I recommend including more recent studies to ensure the literature review is up to date and reflects current research trends.

Minor Issue: A thorough review of the reference list is needed to standardize the formatting, including ensuring consistency in DOI usage, punctuation, and page

numbers. This will improve the professionalism of the references and ensure compliance with the journal's formatting requirements.
