# Supplementary material for: Cultural stigma, psychological distress and help-seeking: Moderating role of self-esteem and self-stigma in Lebanon
Source: PLoS One. 2025 Sep 25;20(9):e0315063. doi: 10.1371/journal.pone.0315063 (PMC12463265; doi:10.1371/journal.pone.0315063)
Supplement: Attachment — Submitted filename: Peer Review Report 20250523.pdf [file pone.0315063.s006.pdf]

Peer Review Report

Manuscript Title: Cultural Stigma, Psychological Distress and Help-Seeking: Moderating role of Self-esteem and Self-stigma in Lebanon

Date of Review: May 23, 2025

Reviewer Comments:

First, I would like to thank the author team for their response to previous review comments and the revisions made. It is evident that efforts have been made to improve the quality of the manuscript, such as deepening the background, refining the description of the methods section, and correcting some minor issues. These positive changes are acknowledged.

However, upon a further detailed review and comparison of Revision 2 - Second revised manuscript with the previous Revision 1- First revised manuscript and the Original Submission-Original manuscript, I have noted some critical issues that were perhaps not fully uncovered in previous reviews, or whose deeper implications merit further emphasis. If these issues are not fundamentally addressed, they will challenge the reliability of the study's conclusions and its overall academic rigor.

This study explores the complex relationships between cultural stigma, psychological distress, self-esteem, self-stigma, and help-seeking behaviors among young adults in Lebanon, a topic of significant practical and academic value. However, the current presentation of data and the articulation of some core research elements make it difficult to accurately assess the authenticity and validity of the research findings.

Major Issues

Q1:Regarding the Consistency between the Reported Sample Size and the Actual Basis for Data Analysis

The manuscript, across different versions and sections (e.g., the “Participants” subsection of the Methods and the title of Table 1), has revised the final sample size from N=245 to N=243, stating the removal of 2 over-age participants and 14 incomplete responses. However, a careful examination of the data details within the manuscript reveals some concerning signs. For instance, in Table 1, the sum of N-values for “Sex” and “Education” categories still amounts to 245 participants, and their percentage calculations also appear to be based on N=245. More critically, the degrees of freedom (df) for t-tests and ANOVAs in Table 2 consistently point to an

analytical sample base of N=245 (e.g., the t-test for "Sex" has df=243, implying N=245; the ANOVA for "Education" has df2=241, which for 4 groups implies N=245). Furthermore, some textual descriptions in the abstract and results section still refer to 245 participants.

Concurrently, the description of the sample exclusion process in the Methods section ("The final sample consisted of 243 participants...14...excluded...2...removed from subsequent analyses.") could be clearer regarding how the final analytical sample of 243 was precisely derived from an initial pool of participants. The current phrasing (e.g., "removed from subsequent analyses") and the lack of explicit mention of the initial total number of individuals before these exclusions might lead to reader queries about the completeness and precision of the screening process.

If the actual statistical analyses were conducted on a sample size inconsistent with the finally reported one, this would constitute a fundamental issue of data accuracy, directly affecting the validity of all descriptive and inferential statistical results**. It is strongly recommended that the authors: Clearly define a unified, final sample size for all analyses.** Based on this sample size, thoroughly re-clean and re-analyze all original data. Subsequently, comprehensively update all relevant figures and statements in the manuscript (including N-values, percentages, means, standard deviations, degrees of freedom, test statistics, p-values, regression coefficients, confidence intervals, etc., in text, tables, and figures) to ensure complete data consistency and accuracy throughout. Please detail, clearly and unambiguously, each step of the participant screening process from initial recruitment to final inclusion in the analysis, along with corresponding participant numbers, in the Methods section.

Q2:Regarding the Appropriateness of the Model Fit Index in Moderation Analyses (Table 4)

I noted that in Table 4 (Moderation analyses results) of the current manuscript, the authors report "Nagelkerke R^2" as the model fit index (e.g., "Model 1: Self-esteem as the moderator (Nagelkerke $R^2 = 0.437$; $F = 18.15$)").

Typically, Nagelkerke R^2 (or Pseudo R-squared) is a common metric for assessing the goodness-of-fit of Logistic Regression models, where the dependent variable is categorical. However, judging from the description of the dependent variable "attitude help seeking" in the manuscript (e.g., mean and standard deviation of 2.26 ± .74 in Table 1, and its description as "considered normally distributed" in the statistical analysis section), it appears to be treated as a continuous variable. The PROCESS

MACRO used for moderation analysis, when dealing with continuous dependent variables, is typically based on linear regression models. The Beta coefficients, t-values, and p-values reported in Table 4 also align with the characteristic output of linear regression.

If a linear regression model was indeed used, reporting Nagelkerke R^2 could mislead readers in their judgment of the model's explanatory power and would reflect imprecision in statistical methodology selection and reporting. **It is recommended that the authors: Carefully verify the specific model type used in their moderation analyses and the nature of its dependent variable.** If it is indeed a linear regression model for a continuous dependent variable, the Nagelkerke R^2 reported in Table 4 should be replaced with the standard R^2 (coefficient of determination) or Adjusted R^2, and ensure the F-value reporting is consistent with the chosen R^2 type and model.

Q3:Regarding the Clarity and Accuracy of Research Hypothesis Formulation

The research hypotheses H1, H2, and H3, as listed in the current manuscript, could be further optimized to enhance their normative value and clarity as scientific hypotheses.

For instance, H1's phrasing "stigma contributes to increased psychological distress" carries strong causal implications, which might be overly assertive for a cross-sectional study where correlational language is often more cautious.

H2's phrasing "We mostly posit that... self-esteem, could moderate..." appears somewhat tentative. More critically, this hypothesis (self-esteem moderating the relationship between stigma and self-stigma) does not seem to align perfectly with the moderation analysis actually conducted and reported in the manuscript (Table 4 Model 1 tests self-esteem as a moderator of the relationship between psychological distress and help-seeking attitude).

H3's formulation "This could be accounted for because..." reads more like an explanation or an inference for a phenomenon rather than a directly testable predictive statement.

The statement of research hypotheses should strive for clarity, precision, testability, and strict alignment with the research design and the actual data analysis methods employed.It is suggested that the authors rephrase the hypotheses with more direct and affirmative language. For cross-sectional studies, prioritize using terms that describe relationships between variables. Crucially, ensure that H2 aligns with the

moderation model actually analyzed or that analyses testing the original H2 are supplemented. H3 might be reformulated as a more direct relational prediction or its logic integrated into the theoretical exposition of other hypotheses.

Q4:Regarding the Reporting and Interpretation of Psychological Distress (Kessler K6) Scores

The “Measures”section of the manuscript states that the Kessler scale total score ranges from 0-24, with a score ≥13 indicating higher distress. However, Table 1 reports the sample's mean psychological distress score as 3.06 (SD=0.93). If this is indeed a total score on the 0-24 scale, it suggests a very low average level of distress for a sample described as “diagnosed with at least one mental illness.” The x-axis range for psychological distress in Figures 1 and 2 (approximately 2.13 to 3.99) also seems to reflect this lower score range, which adds to the interpretative challenge.

The authors urgently need to clarify in the “Measures” section and in the notes for Table 1 whether the reported K6 psychological distress score is a total sum score or a mean item score. Based on this clarification, it is imperative to re-evaluate whether the mean score in Table 1 is consistent with the sample characteristics and ensure that all subsequent delimitations and discussions of “high/low psychological distress” in the manuscript align with this clarification.

Minor Issues

Q1:Regarding the Pervasiveness of Language Strongly Implying Causality

Despite the authors' previous indication (in response to R2 review point 5.2) that causal language would be revised, a thorough reading of the current manuscript’s Abstract, Background, Discussion, and even Conclusion sections still reveals numerous instances of phrases with strong causal implications (e.g., “leads to”, “results in”, “causes”, “worsens”, “degrades”, “shapes”, “contributes to”, “impacts”, ...). These terms, when describing relationships between variables, carry varying degrees of causal directionality. For a cross-sectional study design, which has inherent limitations in inferring causality, such expressions can easily lead to an over-interpretation of the findings.

The authors should undertake a systematic review of the entire text to revise all statements with strong causal implications, replacing them with phrasing more appropriate for describing associations or correlations found in cross-sectional

research (e.g., consider using “is associated with,” “is related to,” “correlates with,” “is linked to,” “may correspond with,” “is observed alongside”). This revision should be consistently applied throughout the manuscript to ensure the rigor of the research report and the accuracy of its statements.

Q2:Accuracy of Percentage Summation in Tables

In Table 1, the sum of percentages for “Age” categories (99.2%) and “Education” categories (100.4%) do not precisely equal 100%. The sum of percentages for types of mental illnesses in the Results text exceeds 100% (110.5%).

Please carefully check the calculation and rounding of percentages in Table 1 for each category to ensure their sum is within a reasonable rounding error of 100%. For the types of mental illness, if multiple diagnoses are allowed, leading to a sum >100%, this should be explicitly stated in the text to avoid reader misinterpretation.

Q3:Interpretation of Self-Stigma Scores in the Discussion

The Self-Stigma Questionnaire (SSQ) is scored such that “higher scores indicating lower levels of self-stigma”.

When discussing the moderation effects in Table 5 related to low SSQ scores (e.g., 1.27), it is advisable to explain more clearly and consistently that this represents a state of “high self-stigma” (at the construct level) to help readers accurately grasp the direction and meaning of the moderation effect.

Q4:Accuracy of Statistical Table Details

The degrees of freedom (df1=3) for the Age ANOVA in Table 2 appear inconsistent with the three age groups listed in Table 1 (if three groups were actually compared, df1 should be 2).

Please verify the actual number of groups used in the ANOVA for Age and ensure that the reported degrees of freedom in Table 2 accurately reflect the analytical design.

Overall Conclusion and Recommendations:

Considering the severity of the major issues identified, particularly regarding the fundamental discrepancy between the data analysis basis and the reported sample size, the inappropriate reporting of the R^2 statistic, the problematic formulation and mismatch of research hypotheses, critical questions about the interpretation of psychological distress scores, and the pervasive use of causal language, I recommend that this manuscript requires ***Major Revision***.

Only after these critical data and core research element issues are thoroughly

addressed, and all analyses are based on a clearly, consistently, and accurately reported sample size using appropriate statistical reporting methods, can the scientific merit and reliability of this study's conclusions be more accurately assessed. The current state prevents the reviewer from fully trusting the presented results and the study's logical and methodological soundness.
