# Supplementary material for: Cultural stigma, psychological distress and help-seeking: Moderating role of self-esteem and self-stigma in Lebanon
Source: PLoS One. 2025 Sep 25;20(9):e0315063. doi: 10.1371/journal.pone.0315063 (PMC12463265; doi:10.1371/journal.pone.0315063)
Supplement: Attachment — Submitted filename: Peer Review Report 20250609.pdf [file pone.0315063.s007.pdf]

Peer Review Report

Manuscript Title: Cultural Stigma, Psychological Distress and Help-Seeking: Moderating role of Self-esteem and Self-stigma in Lebanon

1. General Comments

The authors have responded to previous reviewer comments in this revision, and the manuscript has improved in terms of data consistency and the alignment between hypotheses and analyses. The study's topic, concerning mental health stigma and its correlates in the Lebanese cultural context, is of research value.

However, for the manuscript to meet the standard for publication, several issues still require attention. Revisions are recommended to further enhance the manuscript's scientific rigor, clarity, and accuracy in its interpretation, methodological reporting, and language.

2. Specific Issues for Correction

2.1. Rigor in Interpretation and Language

Correction of Causal Language: The study employs a cross-sectional design, which does not support causal conclusions. However, the text frequently uses language that implies causality (e.g., "leading to" in the Abstract; "deters" and "exacerbating" in the Discussion), which constitutes an over-interpretation of the findings. It is recommended that the authors systematically revise the manuscript to replace such terms with more precise, associative language (e.g., "is associated with," "is related to") to ensure the conclusions are rigorously supported by the data.

2.2. Accuracy in Methods and Results Reporting

2.2.1 In the 'Participants' subsection of the Methods, the sentence "The number of participant responses that were excluded from the analysis was 14 due to incomplete survey responses." is repeated verbatim. One instance should be removed.

2.2.2 In the Results section, the sum of the percentages for different mental illness diagnoses exceeds 100%. This suggests participant comorbidity, but this is not explicitly stated. A brief explanatory note should be added to clarify this for the reader.

2.3. Language and Formatting Corrections

2.3.1 Spelling Errors:

- In the 'Focus on the Lebanese Context' subsection, "loosers" should be corrected

to “losers”.

- In the ‘Limitations and Future Research’ subsection, “ofour” should be corrected to “of our”.

2.3.2 Grammar and Punctuation:

- In the ‘Participants’ subsection, there is a stray space before the period in “researchers .”.
- In the description of the Self-Stigma Questionnaire SSQ, the phrase “high level of internal consistency, and Cronbach's alpha coefficients ranging from 0.75 to 0.901.” is a sentence fragment and should be corrected.

2.3.3 Phrasing and Word Choice:

- In the Discussion, “complexified” is non-standard and should be replaced with “complicated”.
- In the Statistical Analysis section, “adjusted over the following covariates” is unidiomatic. The standard preposition is “for.”
- The phrase “The student t” is informal. It should be revised to the more standard “A Student's t-test.”

3. Final Recommendation

The core research presented in the manuscript is sound. However, the manuscript requires a thorough proofreading to address a number of basic errors before it can be considered ready for publication. **I strongly recommend that the authors conduct a comprehensive review of the entire text to correct all errors in grammar, spelling, punctuation, and capitalization, including the specific examples provided in this report.**

Once these essential corrections have been made, the manuscript will represent a valuable contribution to the literature. Therefore, my recommendation is as follows: ***Minor Revision.***
