# Supplementary material for: Cultural stigma, psychological distress and help-seeking: Moderating role of self-esteem and self-stigma in Lebanon
Source: PLoS One. 2025 Sep 25;20(9):e0315063. doi: 10.1371/journal.pone.0315063 (PMC12463265; doi:10.1371/journal.pone.0315063)
Supplement: Attachment — Submitted filename: Peer Review Report 20250625.pdf [file pone.0315063.s011.pdf]

Peer Review Report

To the Editor and Authors,

Thank you for the opportunity to review this revised manuscript. I would like to begin by commending the authors for this important and timely study. Research that gives visibility to what you've rightly called an "underrepresented part of the world" is essential for a truly global scientific discourse, and your work is a valuable contribution to this effort.

My comments below are offered in this same spirit of constructive collaboration. The study is on a strong footing, and my feedback is focused on refining the interpretation of the findings to ensure the final paper is as robust and impactful as possible.

1.On the Interpretation of Key Findings

My primary feedback centers on two key areas in the Discussion section where the interpretation appears to be disconnected from the empirical data presented in your results.

1.1The Relationship Between Age and Help-Seeking Attitudes: The discussion of age as a factor in help-seeking caught my attention. **The narrative currently suggests a simpler relationship than the more nuanced picture painted by the data in Table 2.** Specifically, the text in the Discussion section states: "*In our sample, adults in their 30s showed more positive attitudes towards help-seeking than younger adults*" and "*They also showed more positive attitudes than individuals over 30*". **This appears to conflict with the data, where the 40+ age group (Mean = 3.55) actually scores higher than the 30-39 group (Mean = 2.94).** Revisiting this section to accurately describe and explore the complex, non-linear trend present in your data could make for an even more insightful discussion.

1.2The Moderating Role of Self-Stigma: A similar, though more subtle, issue of interpretation arises in the analysis of self-stigma's moderating role—a key finding of the study. The discrepancy seems to stem from the reverse-scoring protocol of the Self-Stigma Questionnaire (SSQ), which is correctly noted in the Methods section ("higher scores indicating lower levels of self-stigma"). The results in Table 5 show that the negative relationship between psychological distress and help-seeking is significant at "low and moderate scores" on the SSQ. Therefore, **the correct interpretation is that the effect is strongest for individuals with high and moderate levels of self-stigma. The Discussion section, however, currently draws the opposite conclusion: "…*individuals experiencing high psychological distress are less likely to seek help if they also have low to moderate levels of self-stigma*".** Rectifying this interpretation is crucial for the scientific accuracy of the manuscript's conclusions.

2.On Reporting Precision

On a smaller scale, a couple of points regarding reporting precision could further polish the manuscript.

2.1Alignment of the Abstract with Results: The Abstract makes a specific and compelling claim that "*fear of individuals with mental illnesses, prejudice and lack of knowledge/awareness were related with internalized stigma…*". However, **the Results section primarily presents analyses of the scales' total scores and does not appear**

to offer a direct quantitative test of these specific components. To maintain strict scientific alignment, the conclusions in the abstract should be directly supported by the analyses presented in the body of the paper.

2.2Minor Data Consistency: **A very minor numerical inconsistency was noted between the text's report of bachelor's degree holders ("had a bachelor degree (75.0%)") and the figure presented in Table 1 (74.7%).** Ensuring perfect consistency in these small details enhances the overall professional quality of the work.

3.A Note on This Detailed Review

I would also like to add a brief note about the detailed nature of this review, especially at a later stage. My intention here is wholly constructive. Peer review is a progressive process; as major issues of framing and methodology are resolved in early rounds, it becomes possible for the reviewer to focus on the finer details that elevate a good paper to an excellent one. The considerable time I have invested in this review reflects my belief in the high value and potential of your research. My goal is simply to collaborate in ensuring this important work is as robust and impactful as possible upon publication.

4.Conclusion

To reiterate, this is important and valuable research. The issues I have outlined are all addressable. I am confident that a revision that addresses these points, particularly the two core issues of data interpretation, will result in a very strong and compelling paper.
