# Supplementary material for: Cultural stigma, psychological distress and help-seeking: Moderating role of self-esteem and self-stigma in Lebanon
Source: PLoS One. 2025 Sep 25;20(9):e0315063. doi: 10.1371/journal.pone.0315063 (PMC12463265; doi:10.1371/journal.pone.0315063)
Supplement: Attachment — Submitted filename: StigmaStudy_ReviewerResponse_170225.docx [file pone.0315063.s004.docx]

**Dearest Editor**

**On behalf of my team, I want to thank you and the reviewers to efficiently invest time to help us uplift our manuscript, all while ensuring scientific rigor.**

**We are hereby submitting our revisited our manuscript, massively shaped by the recommendations of the team of experts. We acknowledge it has helped us further refine our narratives and hope it meets the publication standards**

**As such, we are providing below the point-by-point response to each of the 3 reviewers in bold. We have highlighted changes in text when feasible to facilitate tracking.**

**Looking forward to your feedback**

**Myriam El Khoury-Malhame**

**Reviewer #1:**

The manuscript appears to be technically sound, and the data presented generally support the conclusions drawn. The research design and methods are appropriate, and the findings seem logically derived from the data. The authors have provided enough evidence to back their claims, and the study is consistent with the research questions posed. The statistical analysis is generally well-executed, with appropriate methods used to analyze the data. The authors have presented the statistical results clearly, and the significance of key findings is adequately reported.

**Thank you for the humbling feedback**

However, one area of concern is that the raw data underlying the findings have not been fully made available. According to the PLOS ONE data policy, it is important that all data be accessible unless there are justified restrictions, such as privacy concerns. It would be helpful if the authors could provide the raw data or clarify any issues regarding data sharing.

**Data cannot be shared publicly due to restrictions imposed by the Ethics Committee, but is available upon request. Data requests may be sent to the corresponding author.**

In terms of presentation, the manuscript is written in clear, standard English, and the overall structure is intelligible. The content is presented in a logical sequence, making it relatively easy to follow for readers, and the language is consistent with academic standards.

**Much appreciated comment, thank you!**

**Reviewer #2:**

The manuscript addresses a highly relevant and globally significant topic. Understanding the mechanisms and variables influencing help-seeking behavior in individuals with mental health disorders is crucial. The analyses conducted and the results obtained reflect findings already established in international literature, but the study provides an interesting perspective on the specific context of Lebanon.

**This is indeed the objective of the project, to give visibility to the oriental Levantine side of the Global South.**

While the manuscript addresses a highly relevant topic and contributes to understanding help-seeking behaviors in a specific cultural context, several aspects require substantial revision and clarification to enhance the clarity, rigor, and overall impact of the study. As such, I recommend major revisions before the manuscript can be considered for publication.

I recommend clarifying the geographical or cultural group of interest in the title. This would avoid potential confusion, especially since the manuscript does not include specific measures targeting "cultural stigma," as suggested in the title, making it unclear if the study directly addresses the impact of this construct.

**We have added the geographical group and rephrased the title which now reads: “Cultural Stigma, Psychological Distress and Help-Seeking: Moderating role of Self-esteem and Self-stigma In Lebanon”**

Introduction:

The introduction is comprehensive and effectively outlines the variables studied. Given the relevance of the study to the Lebanese context, the authors could emphasize data specific to this national context earlier in the introduction while reducing details on aspects, such as structural stigma, that are not subsequently addressed in the manuscript.

**Additional data specific to the Lebanese context was added early on in the introduction whereas it was clustered towards the end of the introduction in the previous version. The detailing of structural stigma was also removed as it is indeed not addressed in the manuscript.**

Additionally, the introduction cites a study by El Khoury-Malhame et al. (2024), but the type of trauma referenced is unclear, making its relevance to the manuscript debatable.

**We have clarified the choice of reference**. **The intention was to highlight the finding that stigmatized attitudes towards help seeking are massively preventing people from reaching out to mental support, even in the aftermath of an acute trauma, with ongoing outreach campaigns and availability of funding for mental health initiatives in the country.**

Finally, most references are relatively dated, with only a few recent sources. I suggest incorporating more up-to-date references.

**Thank you for the mention. The team has completely revisited its references and has updates them systematically.**

Methods
The measures section is detailed but omits some important information. For instance, some instruments lack a description of the scoring range, making it difficult to interpret the results.

**We have added the omitted information when feasible to make the results easier to interpret. For some scales, there was no defined cutoffs, we nonetheless have highlighted the directionality of scores, for instance with higher scores indicating higher stigma.**

Additionally, two instruments measure stigma, but the distinction between their constructs is unclear (e.g., what specific type of stigma does the The Stigma Scale measure?). Listing these two instruments consecutively could improve the paragraph's readability.

**Thank you for the mention. We have highlighted the crucial yet understandably subtle differences between:**

**- Stigma scale: measuring overall stigma around mental health**

**- Self-Stigma scale: measuring the internalized stigma of one’s own mental illness**

**Scales were also listed consecutively as suggested.**

In the statistical analysis section, there are placeholders marked as "(ref.)" where references are missing.

**This has been amended and the empty ref. were removed. Apologies about the mishap.**

Attention should also be given to the formatting of statistical significance (e.g., uppercase or lowercase "p").

**This has also been amended and fixed throughout the manuscript.**

Results
The results section raises some concerns. The age distribution is heavily skewed towards the 20–29 age group, with only two participants in the oldest age group. This discrepancy makes it challenging to consider the groups comparable. Revisiting the age classification to create more balanced groups might be helpful.

**The purpose of the study as stated in the introduction was to assess stigma and self-stigma in young adults to avoid inter-generational challenges when it comes to understanding and awareness or acceptability of mental health stigma.** **In line with this, only individuals 18-40 were included and the 2 “older” participants were removed to avoid skewing the results.**

**This has been added to the limitation section as well to better investigate potential variability of results with older age groups.**

A similar issue arises with the education levels, where bachelor’s and master’s degree holders constitute the vast majority of the sample. Table 2 reflects these disparities, and the unequal group sizes could undermine the statistical comparisons.

**As per the above, we have acknowledged this and added the limitations related to both age and education in the limitation section**

It would also be helpful to specify which age or education groups differ significantly from one another rather than only providing the general significance level.

**Spot on comment. We added the results of the Bonferroni post-hoc analysis as follows:**

**“The Bonferroni post-hoc analysis results showed that, in terms of age, there was a significance difference between the group aged 20-29 years and the following age groups: 18-19 years (p = 0.002), 30-39 years (p = 0.032) and 40 years and above (p = 0.041). In terms of education, there was a significant difference high school participants and those with a bachelor degree (p < 0.001) and those with a master degree (p = 0.003).”**

For Table 5, it is unclear how the scores were categorized into "low," "moderate," and "high," and the sample sizes for each group should be provided.

**Thank you for this comment. We added this sentence to the statistical analysis paragraph:**

**Interaction terms were probed by examining the association of one predictor with the dependent variable at the mean, 1 SD below the mean and 1 SD above the mean of the moderator.**

**A footnote was also added below Table 5 explaining the above idea.**

Discussion
The discussion is detailed but occasionally reads like an introduction, as it focuses on external studies rather than interpreting the manuscript’s findings. For instance, the paragraph on "Help-seeking in relation to stigma and psychological distress" includes studies that might be better suited to the introduction.

**Thank you for the suggestion. We have added focus on results analysis and discussion, shifted some references as per the recommendation to the introduction and rephrased or removed redundant explanations. All the remodeling was highlighted for ease of tracking.**

The authors note that older age and higher education levels are protective factors for help-seeking attitudes. However, the data do not fully support this claim. For instance, the 18–19 age group scores higher than the 20–29 group and similarly to the 30–39 group. Revising this paragraph to focus on interpreting the results and exploring possible reasons for the observed age and education distributions would strengthen the discussion.

**Thank you for the keen observations. We have attempted to discuss this non-linear relationship, highlighting nonetheless the impact of education and age on the overall framework in the Lebanese context**. **The manuscript now reads “Education and older age could play important yet variable roles in favor of help-seeking behaviors... to be acknowledged and treated”.**

In the paragraph on "Help-seeking in relation to self-stigma," the authors define stigma as affecting students and their families, but this narrowing of the construct should be clarified—why are the authors only considering students? Does it refer to some characteristics of the sample which are not fully described in the method section?

**Apologies for the confusion. The intent was to mention young adults and not merely students and the proper description has been amended as such.**

Furthermore, it is unclear why participants with lower self-stigma are less likely to seek help. This counterintuitive result warrants further discussion, but instead, the authors argue that higher self-stigma increases reluctance to seek help, which is not supported by the results. I suggest rewriting this section to focus on interpreting the findings and exploring their implications.

**Thank you for the comment. We have attempted to increase clarity by reformulating the statements and mentioning nonetheless that “Additionally, in our research, the interaction effect between self-stigma and psychological distress underscores the complexity of help-seeking behaviors”**

Limitations
The authors could explicitly address the uneven distributions in age and education, which complicate inferences about these variables.

**This has been done accordingly. We have added mentions about that “It could also be beneficial to compare findings to those in other age groups: either younger adolescents and/or older adults to better assess the evolution of stigma around mental health as a function of developmental as well as generational, cultural and educational factors. Our study indeed focused on educated young adults and should thus be generalized with care. Future research could also focus on refining the role of education in mitigating or inflating stigma and help-seeking by comparing various levels of education and higher education.”**

Conclusion
The authors could expand on the potential implications and impact of their findings on the Lebanese context, which would strengthen the significance of the manuscript.

**This has been done accordingly**

Minor points: There are a few typographical errors throughout the manuscript (e.g page 15: "developped", page 19: "as shown" instead of "has shown") that should be carefully reviewed and corrected. A thorough proofreading is recommended to ensure consistency and accuracy in the text."

**Thorough proofreading has been done, hopefully to the standards of the journal.**

**Reviewer #3**

Main Issues, Arguments, and Conclusions

This study explores the connections between Cultural Stigma, Psychological Distress, and Help-Seeking Behavior, with a particular focus on the moderating roles of Self-esteem and Self-stigma. Using a cross-sectional online survey of 245 young Lebanese adults, the study found significant negative associations between cultural stigma and both psychological distress and help-seeking attitudes. Moreover, self-esteem and self-stigma affected these relationships, increasing psychological distress and reducing help-seeking intentions when cultural stigma was high and self-esteem was low or self-stigma was high.

The findings highlight that the harmful impact of cultural stigma on mental health is particularly strong in the Lebanese cultural context. The moderating roles of self-esteem and self-stigma provide important insights for designing culturally relevant mental health interventions. The study suggests that mental health interventions should consider cultural differences, focusing on improving self-esteem and managing self-stigma to help individuals cope with stigma-related distress and increase their willingness to seek help.

Positioning and Contributions in the Existing Literature

This study adds value to the existing literature by examining the dynamics of cultural stigma, psychological distress, and help-seeking behavior within the specific cultural context of Lebanon, a non-Western setting that has been underrepresented in mental health stigma research. Unlike much of the existing literature based in Western contexts, this study makes the following key contributions. First, it broadens the cross-cultural perspective on mental health stigma by examining how cultural factors shape stigma and its consequences in Lebanon’s social and cultural environment. The empirical data from this non-Western context enhance cross-cultural comparisons in this field.

Second, the study improves our understanding of how self-esteem and self-stigma influence the effects of cultural stigma on psychological distress and help-seeking behavior. This theoretical insight supports the use of moderation effects when designing targeted mental health interventions.

Finally, the study provides practical suggestions for tackling cultural stigma, particularly for groups with high levels of stigma. By emphasizing the importance of strengthening self-esteem and reducing self-stigma, the study offers useful guidance for policymakers and mental health professionals who aim to design culturally appropriate interventions.

Strengths and Weaknesses

This study has several strengths. It addresses an important issue by exploring how cultural stigma affects mental health and help-seeking behaviors, with a focus on the moderating roles of self-esteem and self-stigma. This focus enhances our understanding of the psychological processes involved in mental health and offers valuable insights for future research and interventions. The study also uses well-established psychological measurement tools, such as the Rosenberg Self-esteem Scale and the Kessler Psychological Distress Scale, to ensure the accuracy and reliability of the data. Furthermore, the study contributes to the broader field of cross-cultural research by exploring cultural stigma in a non-Western context, specifically Lebanon, which fills a gap in the literature.

However, there are areas for improvement. One limitation is the representativeness of the sample. The study primarily focuses on young adults aged 20-29, which may limit the ability to apply the findings to other age groups or social demographics. Additionally, the reliance on online recruitment via social media platforms may introduce bias, narrowing the diversity of the sample.

Another limitation lies in the theoretical analysis of the moderating mechanisms. While the study identifies the roles of self-esteem and self-stigma as moderators, it does not explore these effects in enough detail. This lack of explanation means that the reader does not fully understand why or how these moderators affect the relationships between cultural stigma and mental health outcomes.

Finally, the study could go deeper into the specific cultural factors in Lebanon, such as family values and social norms, that influence the development and persistence of cultural stigma. A more detailed exploration of these cultural factors could provide richer insights into the broader implications of the findings.

**Thank you for taking the time to engage with and summarize important key aspects of the study. This is personally heartwarming and professionally rewarding.**

General Recommendations

To improve the quality and impact of the research, several suggestions are made. First, expanding the sample to include a wider range of participants, such as those from different age groups and professions, would improve the applicability of the findings.

**Thank you for the suggestion. The target of the study was to study a young adult population, considered to be more homogenous from a generational perspective including sociopolitical and economic situation, as well as parenting style and trends. Participants were included between 18-40 years and thus the 2 older participants skewing the results were removed.**

**We have also added a mention in the limitation section as to the need to include, perhaps in future ventures, a wider range of participants.**

Second, a more thorough theoretical analysis of the moderating mechanisms would strengthen the study’s conceptual foundation. By drawing on established theories, such as stigma management theory or social cognitive theory, the study could better explain the internal logic behind the moderating effects observed.

**We are absolutely delighted with the reminder to include a theoretical framework to better explain the results and as such we have done just that at the end of the discussion, before the limitation. The manuscript now reads “Study results can be mapped to the overall stigma management theory which posits that individuals facing stigma engage in various coping strategies, such as passively concealing, covering, minimizing the stigma or actively confronting it to dampen its negative impacts (Meisenbach, 2010). Within this framework, self-esteem could be viewed as a buffer against cultural stigma whereby individuals with higher self-esteem and self-affirmation could maintain a positive image despite societal disapproval, and by subsequently mitigating their psychological distress they would be more likely to seek support. On the contrary, self-stigma would amplify cultural stigma and individuals who internalize social prejudice would alternatively partake in fear of judgment and avoid challenging cultural beliefs, therefore experiencing heightened shame and self-blame leading to increased psychological distress and decreased help-seeking.”**

Third, incorporating a detailed examination of Lebanese cultural norms and their role in shaping stigma would enhance the cultural analysis and strengthen the study’s cross-cultural contributions.

**Done as per the recommendation.**

Fourth, the study could benefit from more specific and actionable recommendations for interventions. These could include educational programs aimed at improving self-esteem, reducing stigma, and raising mental health awareness through community campaigns.

**We have added this to the recommendations at the level of the conclusion.**

Fourth, increasing transparency in the methodology, particularly regarding participant recruitment and data collection processes, would improve the study’s credibility and replicability.

**This has been amended as per recommendation.**

Lastly, improving the clarity and consistency of the language throughout the paper is essential. The English language expression, including punctuation, grammar, and overall writing style, should adhere more closely to academic norms for journal publications.

**Thank you for the mention. The linguistics have been properly reviewed by a native English speaker to raise to standards.**

In summary, this study offers valuable insights into the dynamics of cultural stigma, self-esteem, and self-stigma in a non-Western cultural context. While it successfully identifies key relationships and provides practical recommendations, addressing the limitations in sample representativeness, theoretical depth, and cultural context would

significantly enhance its contribution to the field. The study holds great potential to inform culturally sensitive mental health interventions and inspire further research in cross-cultural psychology.

**Again, we hereby express our gratitude for acknowledging the importance and relevance of the topic, all while adding invaluable scientific rigor. Thank you**

Examples and evidence

**These recommendations have been systematically reviewed and actioned. We thank you for taking the time to closely monitor the very many aspects of research and manuscript production and relay the comments in constructive ways.**

Literature review section

Major Issues

(1)The literature review section is not grounded in a clear theoretical framework, resulting in insufficient theoretical depth throughout the review. It is recommended that the author use relevant theories to strengthen the relationship statements between the variables of interest, thus laying a more solid theoretical foundation for proposing the hypotheses of this study.

**Much needed input. This has been done in reference to the suggested stigma management theory both in the introduction and discussion segments.**

(2) Lack of specificity in describing the research gap. Although the manuscript points out that "research on the influence of stigma on mental health and help-seeking behavior is scarce" in Lebanon, it fails to specify the limitations of existing studies (e.g., Wehbe, 2011; Hassan, 2015), which affects the positioning and value of the current research.

**Limitations of existing studies have been mentioned and highlighted as recommended. The segment now reads “Existing studies have almost exclusively centered on student population, and did not focus enough on personal qualities such as self-esteem, which could potentially offer important intervention value to counter the impact of stigma.”**

(3) Overly broad statement of research objectives. The manuscript mentions at the end that "The purpose of this research is thus to provide empirical data on the influence of stigma on self-esteem and help-seeking behaviors," but it does not propose specific research questions or hypotheses.

**The research objectives have been framed withing the stigma management theory framework and the hypotheses have been clearly stated as per the suggestion. These changes are highlighted in text to facilitate tracking.**

(4) Absence of research hypotheses: The literature review section does not explicitly state research hypotheses, although they can be inferred from the title and research content (e.g., the moderating roles of cultural stigma, self-esteem, and self-stigma). The lack of clear research hypotheses may result in a lack of theoretical support and explicit hypothesis testing in the results section, potentially leading to a more descriptive rather than hypothesis-driven study.

**As per above, we have clearly mentioned research hypothesis to reflect the value of this investigative study.**

Minor Issues

(1) Regarding structure, the transition from global issues to the specific situation in Lebanon is not smooth enough. It is suggested to use headings to structure this section and enhance its logical flow.

**Section Headings have been added to facilitate flow as recommended.**

(2) Regarding data, the WHO (2019) data needs to be updated, and the data stating that "75-80% of people with mental disorders not receiving therapy" also requires a more recent source.

**As per reviewer #2 recommendations, all outdated references have been systematically reviewed. The WHO reference is more of a citation around stigma than data and as such have been left unchanged.**

(3) Regarding local studies, the discussion of the Lebanese situation mainly relies on research by Karam et al. (2019) and Hassan (2015), and more recent local studies need to be added.

**Interesting point. This is indeed why we mentioned the scarcity of research, notably with the protracted crises in the region that have probably shifted research towards more direct assessment of predictors of mental distress and interventional studies as opposed to prevention ones, looking into cultural aspects of stigma that are perhaps more challenging to impact (as opposed to suggesting for instance targeting interventions for those more vulnerable to trauma).**

(4) Regarding writing expression, the use of the tense "has been recently investigated" does not match the cited 2009 study (Quinn & Chaudoir). From a cross-cultural perspective, the discussion of "global South and Arab countries" lacks a systematic comparison with other cultural contexts.

**Thank you for the mention. In the general reformatting of the manuscript as per reviewers’ recommendations to increase clarity and readability, we have provided**

- **a theoretical framework**
- **a precise understanding of the local context**

**both introduction and discussion were massively reshuffled, and we do hope this has properly addressed the raised concern.**

(5) Reliance on a single source for defining core concepts. The definition of social stigma in the manuscript only cites Watson et al. (2003): "social stigma refers to the public beliefs and impressions regarding a specific person or community," lacking support and in-depth discussion from more recent literature.

**The authors have chosen one source, in addition the WHO to define the constructs and focus of the current study. It will be indeed very interesting in perhaps upcoming manuscripts to further lead in-depth discussion of the literature. The idea was to start laying foundation on the matter of social stigma in Lebanon and other Levantine countries before engaging in thought-provoking debates.**

(6) Updating references: The overall literature in the review section needs to be supplemented with the latest literature relevant to the research topic.

**Done.**

Methods section

Major Issues

(1) The data collection period "between March 2023 and August 2024" is excessively long, and the actual data collection time frame needs to be clarified. An unusually long data collection period may affect the temporal consistency of the data, introduce potential time-effect biases, and indicate potential issues with the study execution process.

**Thank you for the comment, the data was actually practically collected in 2023 and later analyzed in 2024 but since the google link remained active, we have reported it as such.**

(2) The manuscript mentions, "Participants responded to the survey link shared via social media platforms such as Instagram and WhatsApp." The specific processes for participant recruitment and questionnaire distribution need to be clearly described: Were the questionnaires sent to pre-identified eligible participants via social media, or was recruitment conducted openly on social media? This affects readers' understanding of the research methods and their judgment of the reliability of the results. The current content only lists the participant inclusion criteria and the number of excluded questionnaires, completely lacking a substantive description of the study execution process. This section should clearly state "how the study was conducted." It is recommended to further clarify how the research team reached potential participants, how participants were recruited, how the questionnaires were distributed, how data were collected, and what the entire research process was like. The absence of this key information makes it impossible to assess the repeatability and methodological quality of the study. The authors are advised to provide a complete description of the study execution process, rather than merely listing the inclusion criteria and exclusion numbers.

**We have addressed the concern to the best of our abilities. Research assistants on the project indeed circulated the link on their social media accounts, which is a preferred way of communication in the country, without necessarily targeting eligible participants. Snowballing effect was encouraged as well.**

(3) There are several apparent issues in the "procedure" section of the manuscript. First, although the manuscript states, "Only participants who were above 18, living in Lebanon and diagnosed with at least one mental illness were eligible to participate," it does not further specify the diagnostic categories or present the distribution of specific mental disorder diagnoses among the participants. This lack of detailed diagnostic information may affect the interpretation of the study results.

**We have added this sentence in the Results section to answer to the distribution “Moreover, 60.0% of our sample had anxiety and stress disorders, 10.6% OCD, 6.5% neurodevelopmental disorders, 5.7% sleep disorders, 22.0% mood and personality disorders and 5.7% other psychiatric problems.**

Second, there is ambiguity in the exclusion of invalid data in the data analysis: Although the article mentions that 14 participants were excluded due to incomplete questionnaires, the Study Design and Sample section still mentions a sample of 245 participants. This statement may cause ambiguity, and readers are unclear whether the final number of participants used for data analysis is 245 or 231 (the number after excluding invalid data). If the data analysis still used 245 participants (including invalid data), it would affect the accuracy of the results, as invalid data may lead to bias and affect the reliability of the conclusions.

**This confusion has been clarified and the section was revisited to clearly introduce procedure and participants subsections. “The final sample consisted of 243 participants”. We have explained the reasons for removing the 14 incomplete data points and additionally the 2 participants over 40 years.**

Minor Issues

(1) There is an important lack of clarity in the description of the Self-Stigma Questionnaire (SSQ). In the current description, there is confusion regarding the source of the scale and the purpose of the literature citation. Specifically, the text describes this 14-item scale and cites Ochoa et al. (2015) at the end, but fails to clearly distinguish the following key information: Who developed this scale? If it was developed by Ochoa et al., it should be explicitly stated when introducing the scale; if not, the original developers should be mentioned. What exactly is the study by Ochoa et al. (2015): the original scale development literature, or merely used to support the argument that "the scale has good reliability and validity across different populations and languages (Cronbach's alpha ranging from 0.75 to 0.901)"? This ambiguous citation style makes it difficult for readers to trace the original source of the scale and determine the specific role of the cited literature. The authors are advised to revise this content to clearly distinguish between the development source of the scale and subsequent validation studies, which is a basic requirement for the description of measurement tools in the methodology section.

(2) The manuscript mentions, "The attitude help seeking score was considered normally distributed since the skewness (= .001) and kurtosis (= -.702) values varied between -1 and +1 [ref]." The authors use skewness and kurtosis values within ±1 as the criterion for judging normal distribution but do not provide a reference to support this judgment criterion. The meaning of [ref] here seems to be not a formatting issue, but a substantive reference that needs to be supplemented.

(3) There are some formatting issues, such as in the sentence "It has been confirmed to be reliable and effective across a range of populations and languages. high level of internal consistency, and Cronbach's alpha coefficients ranging from 0.75 to 0.901 (Ochoa et al., 2015)," where the punctuation is incorrect.

Results section

Major Issues:

(1) It is recommended that the textual description in the results section be expanded. Currently, the narrative is rather brief and overly reliant on tables. The following improvements could strengthen the section:

- Sample Characteristics: The current description simply states, "Two hundred forty-five young adults with diagnosed mental disorders participated in this study." It would be beneficial to provide a more comprehensive description of the sample characteristics.

**As per the recommendations, we added more details as follows:**

**“Two hundred forty-five young adults with diagnosed mental disorders participated in this study. The majority of the participants was aged between 20-29 years (89.8%), was females (60.0%) and had a bachelor degree (75.0%). Full socio-demographic details and the mean/standard deviation of the scores are represented in Table 1.”**

- Bivariate Analysis Results: The existing statement, "The results showed that older participants and those with higher education compared to the other categories had significantly higher help-seeking attitude," is overly succinct. It is suggested to include specific information about the magnitude and patterns of these differences.

**Same as above, we are happy accommodating for the comment and have added the results of the Bonferroni post-hoc analysis as follows:**

**“The Bonferroni post-hoc analysis results showed that, in terms of age, there was a significance difference between the group aged 20-29 years and the following age groups: 18-19 years (p = 0.002), 30-39 years (p = 0.032) and 40 years and above (p = 0.041). In terms of education, there was a significant difference high school participants and those with a bachelor degree (p < 0.001) and those with a master degree (p = 0.003).**

**We also added the effect size values as requested.”**

- Moderation Analysis Results: The current description, such as "The interaction self-esteem by psychological distress was significantly associated with help-seeking attitude (Beta = .08; t = 4.07; p < .001)," leans towards a technical tone. It is recommended to add an interpretation of the substantive significance of these results.

**We added these sentences as suggested:**

**“This suggests that individuals with varying levels of self-esteem may differ in their likelihood to seek help when experiencing psychological distress.**

**This suggests that individuals with varying levels of self-stigma may differ in their likelihood to seek help when experiencing psychological distress.“**

(2) The study identifies two significant moderation effects, and it is recommended that these results be presented visually for clarity. Currently, Table 4 presents the statistical results of the interaction effects (e.g., the interaction between self-esteem and psychological distress: Beta = .08; t = 4.07; p < .001), and Table 5 presents the conditional effects at different levels. To improve the presentation of these findings, it is suggested to create simple slope plots for both moderation models: Model 1: Plot for the moderation effect of self-esteem/ Model 2:Plot for the moderation effect of self-stigma.

**The two figures were added as suggested.**

(3) It is recommended that the authors include a path diagram in the results section to visualize the moderation effect model. The diagram could effectively demonstrate how the two independent variables (Cultural Stigma and Psychological Distress) influence the dependent variable (Help-Seeking Behavior) through the moderating variables (Self-esteem and Self-stigma). Adding such a visual representation in the results presentation would enhance the clarity of the findings, particularly in illustrating the complexity of multivariable interaction models, and help readers better understand the study’s core results.

**The two figures were added as requested.**

Minor Issues:

(1)To further enhance the informative value of the moderation analysis, it is recommended to include additional model fit information. While the current analysis already presents solid evidence for the presence of moderation effects, including regression coefficients (Beta), significance tests (t-values and p-values), and 95% confidence intervals, additional details could be helpful for a more comprehensive understanding. The following supplementary information is suggested:

- Overall Model Fit Indicators: R2 values at each step, the change in R2 (ΔR2) after adding the moderation term, and the corresponding F-change values with their significance levels.

**The Nagelkerke R^2^ and F values were added as requested.**

- Stepwise Regression Information:
- Step 1: Results of the baseline model with only control variables

- Step 2: Results of the model with main effects included
- Step 3: Results of the model with interaction terms added

**We added the requested results as supplementary table 1.**

(2) It is recommended that the table footnotes clearly display all three levels of statistical significance. Although there may not be any instances of *p < .05 in the results, it is standard practice in statistical reporting to include all levels of significance: *p < .05, **p < .01, ***p < .001. Therefore, the table footnotes should be revised to include these complete descriptions of statistical significance.

**Corrected as suggested.**

Discussion Section

Major Issues

(1)The discussion section lacks a clear articulation of the theoretical framework underpinning this study. It is suggested that the authors dedicate a section at the beginning of the discussion to outline the core theoretical framework of the study, clarifying the interrelations of different theories, in order to construct a complete theoretical analysis framework. Specifically, the discussion of the relationships between stigma, self-esteem, self-stigma, and help-seeking intentions is not closely linked to specific theoretical perspectives. The current discussion primarily relies on previous empirical research findings to support the viewpoints, but it lacks a deep analysis of the theoretical logic behind these results. As a result, readers may find it difficult to understand the correspondence and connection between the study's findings and the key theories. It is recommended that the discussion of key findings be tightly integrated with specific theoretical perspectives, using theoretical concepts and propositions to interpret the empirical results, thereby highlighting the alignment between the research findings and theory.

**Thank you for the highlight. This has been done according to suggestion in line with the stigma management theory and the discussion has been completely revisited in an attempt to construct as is mentioned a complete theoretical analysis framework.**

(2) The discussion currently focuses mainly on the implications of the research findings for understanding the relationships between stigma, self-esteem, self-stigma, psychological distress, and help-seeking intentions, as well as how the study adds to and extends existing research. While these discussions are important, the practical application of the research knowledge is not sufficiently addressed. Given that educational level is an important factor influencing help-seeking attitudes, the authors should provide more targeted and actionable recommendations for improving educational practices based on the study’s results. At present, this aspect is relatively vague and underdeveloped. For example, the authors mention that "education on mental health literacy and service availability is also needed to increase help-seeking behavior," but do not provide specific suggestions on how to carry out targeted educational interventions.

**Spot on feedback. This was really helpful is reframing the overall impact of the manuscript, and, as a result of the previous reshuffling, the implications for the search findings at the personal and the social level have been better elicited notably towards the end of the discussion and in the conclusion section.**

Given that young people are typically in higher education or vocational training, it is particularly necessary and urgent to discuss how the findings could be applied to mental health curricula in schools, the provision of mental health services, and student counseling services. If the authors could offer practical and feasible recommendations for educators and policymakers, this would significantly enhance the practical value of the research.

**This has been done according to suggestions and practical recommendations were added to “In a collectivist Lebanese context, this means capitalizing on social connectedness by intensifying early awareness within appropriate cultural norms. Other actionable steps would include actively fostering youngsters’ self-esteem within families, societies as well as schools, universities and municipalities with specific evidence-based programs aimed at improving self-esteem and reducing stigma. One such program was shown to significantly enhance skills such emotional intelligence in public high-schools and could additionally target self-esteem and self-stigma (Sanchez-Ruiz et al., 2023).**

Additionally, although the authors mention the cultural context of the study (Lebanon) in the case and background introduction, this context is not sufficiently explored in the discussion of practical implications. Educational interventions addressing mental illness stigma and help-seeking in different cultural settings may require distinct strategies and approaches. If the authors consider the cultural characteristics of Lebanon and offer constructive suggestions on how to adapt educational practices accordingly, the practical guidance of the paper would be further strengthened.

**This has been highlighted at various points throughout the discussion, to focus on the particular cultural implication of the Lebanese society, all while mentioning the generalizability to other similar countries and in other regards to all countries indiscriminately.**

Minor Issues

(1)The logical organization and transitions between different points in the discussion need to be improved. There is a sense that the sections are somewhat disjointed, lacking necessary transitions and connections. It is recommended that the authors adjust the structure of the text to enhance the logical flow between the main findings. One approach could be to first summarize the key results and then provide a detailed discussion of each. Additionally, transitional sentences should be added between different sections to tightly link the content.

**Thank you for the recommendation. The structure of the text has been completely revisited as per the suggestion. All transitional sentences and adjustments were highlighted for ease of tracking.**

(2) The discussion of limitations and future research directions needs to be further clarified. The transition between the two sections should be improved. In addition to pointing out the study's limitations, the authors should analyze how these limitations might affect the interpretation of the results, and use these analyses as a basis for suggesting future research directions. For example, while the sample of 245 participants is adequate for data analysis, it may be too small for studies involving multiple variables and moderation effects. The authors should acknowledge the limitation of the small sample size and explain how it may influence the study's results.

**Done as per the suggestions. We have also added limitations related to education and age.**

Reference Section

Major Issue:

The references section contains several outdated citations that require updating. Notably, core references such as Link & Phelan (2001) and Wright et al. (2000) are cited, which may no longer reflect the most recent developments in the field. I recommend including more recent studies to ensure the literature review is up to date and reflects current research trends.

**Thank you. This has been done thoroughly.**

Minor Issue:

A thorough review of the reference list is needed to standardize the formatting, including ensuring consistency in DOI usage, punctuation, and page numbers. This will improve the professionalism of the references and ensure compliance with the journal's formatting requirements.

**This has been done accordingly. Thank you for the nudge.**
