# Supplementary material for: Cultural stigma, psychological distress and help-seeking: Moderating role of self-esteem and self-stigma in Lebanon
Source: PLoS One. 2025 Sep 25;20(9):e0315063. doi: 10.1371/journal.pone.0315063 (PMC12463265; doi:10.1371/journal.pone.0315063)
Supplement: Attachment — Submitted filename: Stigma_ReviewerEdits_080525.docx [file pone.0315063.s005.docx]

**Dear editor,**

**Thank you and the reviewers for your time investment to uplift the scientific rigor and display of our manuscript.**

**We have amended authors list to better reflect individual involvement in the review process. We have also added an expert to help address the culture-related major comments of the reviewer. We have secured authors alignment to move forward with this change.**

**We have subsequently carefully attended to comments and recommendations and are hereby submitting**

- **an updated version of the manuscript with highlighted tracked changes.**
- **a point-by-point reply in bold font to reviewers’ comments**

**We do look forward to your favorable input**

**On behalf of the team,**

**Dr Myriam EL Khoury-Malhame**

Peer Review Comments

Dear Authors,
Thank you for your careful and thoughtful revisions to the manuscript. It is evident that substantial effort has been invested in improving the paper across multiple sections.

- Your incorporation of a theoretical framework

- Expansion of the literature review,

- Clarification of methodology,

- and inclusion of moderation plots have notably enhanced the overall clarity and contribution of the study.

**Thanks a lot for the support throughout and for the constructive criticism.**

After reviewing the revised manuscript, I find that the majority of the major concerns have been addressed. However, a few important issues remain unresolved or only partially addressed. Below is a summary of these points, organized by manuscript sections and marked by importance level.

1. Introduction / Background Section **1.1** While the narrative logic is coherent, the hypotheses remain embedded within the text. I recommend clearly enumerating them at the end of the Background section (e.g., H1, H2, etc.) for improved clarity and structure.

**This has been done accordingly**

**1.2** Please ensure consistency in terminology. The abstract refers to “self-confidence” whereas the main text consistently uses “self-esteem.”

**Terminology consistency has been checked.**

2. Methods Section **2.1** Procedure description lacks coherence and clarity.While the revised Procedure section includes several relevant elements—such as the survey period, platforms used, and eligibility criteria—the current version still reads as a list of disconnected points rather than a logically structured account of the data collection process. Rather than listing discrete elements, the procedure should be presented as a cohesive narrative, enabling readers to follow the actual implementation of the study step-by-step.

**Thank you for the mention, it is indeed important to us to improve narrative-like procedural reporting. As such we have actioned the below suggestions consistently.**

To improve clarity and replicability, I recommend that the authors present this section as a coherent sequence of steps, clearly addressing:
- Who: Who initiated and managed the survey distribution—was it the primary researchers, trained assistants, or both? **Both**

- Where/How: Where and how was the survey shared (e.g., via WhatsApp, Instagram, LinkedIn)? Were any organizational or institutional platforms involved? **Personal online platforms have been reported. No institutional platform was involved.**

- When: When was the survey accessible (precise start and end dates), and for how long? **This has been reported exactly**

- What strategy: How was participation encouraged? For example, paid advertisement, direct messages, or snowball sampling. In particular, the statement “snowballing technique was encouraged” lacks detail.Please clarify how this was implemented—e.g., were participants explicitly asked to share the survey? Were any prompts, templates, or incentives provided? Was this process monitored or tracked? **This has been clarified in text. Participants were indeed asked to share the link. The link was also sponsored via the social media platform to target Lebanese 18-40 years users. No incentives were provided, and the researchers had no way to track or monitor the snowballing process.**

- Quality control: What steps were taken to ensure data integrity, such as screening for eligibility, controlling for duplicate responses, or ensuring informed consent? **Informed consent was provided prior to filling the questionnaire by reading the ethical considerations and clicking an approve button. Duplicate responses were only tracked using IP addresses as no personal identifiers were requested to preserve anonymity and confidentiality. Eligibility was manually checked to only include participants over 18 residing in Lebanon.**

**2.2** The description of the SSQ still does not clearly distinguish whether Ochoa et al. (2015) is the scale developer or simply cited for psychometric validation. Please clarify the original source of the SSQ.

**Thank you for the attention to detail. We have added the following “. [SSQ] is built on a shorter version of the original 29-item scale (Ritsher et al., 2003).”**

**2.3** The placeholder “[ref]” remains in the statistical analysis section following the skewness/kurtosis normality statement. Please provide a proper reference to support the claim that skewness and kurtosis between -1 and +1 indicate normality.

**This has been amended and the reference has been added Field, A. (2013)**

3. Results **3.1** Table 2 presents informative bivariate comparisons; however, several aspects of its structure and presentation could be improved to enhance clarity and align with reporting standards:

- Structural clarity. The current presentation places group means and F/t statistics in the same row (e.g., under “Age” and “Education”), which may confuse readers as to whether the test statistics apply to individual subgroups or to the overall comparison. It is recommended to visually separate subgroup statistics from overall test results—for example, by indenting subgroup rows, using merged cells, or adding a distinct summary row for the F/t test.

**Sorry for the misunderstanding. The F/t statistics are in a distinct row than the means of the groups.**

| **Table 2. Bivariate analysis of factors associated with attitude help seeking.** | | | | | |
| --- | --- | --- | --- | --- | --- |
| **Variable** | **Mean ± SD** | **t / F** | **df / df1, df2** | **p** | **Effect size** |
| **Age (years)** |  | **8.88** | **3, 241** | **<.001** | **.100** |
| **18-19** | **2.84 ± .43** |  |  |  |  |
| **20-29** | **2.18 ± .73** |  |  |  |  |
| **30-39** | **2.94 ± .57** |  |  |  |  |
| **Sex** |  | **-1.43** | **243** | **.155** | **.186** |
| **Male** | **2.17 ± .67** |  |  |  |  |
| **Female** | **2.31 ± .78** |  |  |  |  |
| **Education** |  | **10.82** | **3, 241** | **<.001** | **.119** |
| **High school** | **3.12 ± .64** |  |  |  |  |
| **Bachelor** | **2.15 ± .77** |  |  |  |  |
| **Master** | **2.37 ± .30** |  |  |  |  |
| **PhD** | **3.40 ± .42** |  |  |  |  |

**Numbers in bold indicate significant p values.**

**Please let us know if you want us to make any modifications. Thank you.**

- Effect size specification. The table includes effect size values but does not specify their type (e.g., Cohen’s d, eta-squared η2). For clarity, please identify the type of effect size used and provide interpretation guidance in a footnote (e.g., η2 values of .01 = small, .06 = medium, .14 = large).

**We added this explanation as a footnote:**

**Effect size refers to Cohen’s d for sex (d values of .02 = small, .05 = medium, .08 = large) and eta-squared η2 (η2 values of .01 = small, .06 = medium, .14 = large) for age and education.**

Addressing these points will improve the table’s clarity and help readers more accurately interpret your findings.You may wish to consult recently published tables in PLOS ONE for formatting consistency and reader accessibility.

**Thank you for your suggestion. We made the changes accordingly.**

**3.2** The response suggests that stepwise regression results were included as “Supplementary Table 1,” but this table does not appear to be included in the revised manuscript or appendices. Please ensure it is attached and referenced properly.

**We apologize for this oversight. We added the following to the results section:**

**Linear regressions and Moderation analyses**

**The results of two linear regressions taking the attitude help seeking as the dependent variable, psychological distress as the independent variable and self-esteem as the moderator and taking the attitude help seeking as the dependent variable, psychological distress as the independent variable and self-stigma as the moderator, can be found in supplementary tables 1 and 2 respectively.**

**4.** Discussion **4.1** While the authors provide some general cultural context about Lebanon, the manuscript would benefit from a clearer discussion of how specific cultural elements—such as family dynamics, religious beliefs, or social expectations—might influence stigma and help-seeking behavior in this context. This need not be an exhaustive anthropological analysis, but even brief references to existing local studies, or examples of how such factors have been observed to shape attitudes in daily life or public discourse, would enhance the cultural grounding of the study.

**Some of those elements were introduced in the background part and the discussion was centered to echo those cultural elements as recommended. This was mostly included in the conclusion segment.**

**4.2** The authors have included some general suggestions related to mental health education. To enhance the applied value of the study, it would be useful to briefly reflect on how these recommendations align with the realities of the Lebanese context. For example, are school-based awareness campaigns, university-level counseling workshops, or community-based mental health sessions feasible given current infrastructure or public attitudes? Even a brief reflection on what is currently possible or lacking would help to ground the study’s implications in the local context, without requiring detailed intervention plans.

**This is an excellent point to gauge feasibility and cultural-relevance of those recommendations. A section has been added as some colleagues have already tested the importance of school-based campaigns and university workshops.**

**5.** Other Minor Issues **5.1** Spelling, grammar, and stylistic issues**.** During the review of your manuscript, I've identified several spelling errors and unclear expressions that should be corrected before final submission. Below are specific examples:

- In the "Procedure" section:"...which are preferred common means of communication in Leabanon." → Please correct the country name spelling to "Lebanon"

- In the description of the "Rosenberg Self-Esteem Scale":"It is a 4-point Linkert scale from 0 (strongly agree) to 3 (strongly disagree)."→ This should be corrected to "Likert scale"

- In the "Procedure" section:"...shared via social media platforms of research personal such as WhatsApp..." → This should be revised to "research personnel"

- In the paragraph following "Impact of Stigma on Self-Esteem":"Th negative self-perceptions and lower self-esteem associated with stigma..."→ This should be corrected to "The negative self-perceptions"

- In the "Help-Seeking in relation to Self-Esteem" section: "University students with higher self-esteem seem to be queen of promptly recognizing..." → This expression is unclear and should be rephrased, perhaps as "keen on promptly" or another appropriate wording

- In the discussion section related to stigma management theory:"...they would be more likely to seek support and around it." → This phrase is unclear; please revise to express the intended meaning more precisely .

These revisions will enhance the professionalism and readability of your manuscript. I recommend conducting a thorough language review of the entire document before final submission to ensure no similar errors remain.

**We apologize for the one-too-many mistakes and agree it undermines the quality of the submission. We have reviewed the manuscript thoroughly and hope to have increased professionalism and readability.**

**5.2** Causal language in a cross-sectional study**.** Some expressions (e.g., “X leads to Y”, “results in”) suggest causality. Given the cross-sectional nature of your study, please revise to use correlational phrasing (e.g., “X is associated with Y” or “may relate to Y”).

**Agreed and done.**

Overall, the manuscript presents promising findings and the revised version has addressed many core issues. If the authors can revise the remaining points as suggested above, the manuscript will be significantly strengthened. I therefore recommend Minor Revisions.

**We have recruited a public health expert to upend the cultural aspect in relation to the manuscript as well as to highlight actionable evidence-based recommendations. We have also carefully attended to the minor revisions and do look forward for the publication of this paper.**
