# Supplementary material for: Cultural stigma, psychological distress and help-seeking: Moderating role of self-esteem and self-stigma in Lebanon
Source: PLoS One. 2025 Sep 25;20(9):e0315063. doi: 10.1371/journal.pone.0315063 (PMC12463265; doi:10.1371/journal.pone.0315063)
Supplement: Attachment — Submitted filename: Stigma_PointByPoint_100625.docx [file pone.0315063.s008.docx]

**Dearest academic editor**

**Once again, my team and I are grateful for the timely constructive follow-up**

**Thank you for your patience as we finalize the uplift of our paper. We have hereby addressed the reviewer comments in bold and have highlighted changes in the manuscript accordingly.**

**We do hope these changes make the manuscript suitable for sharing**

**Best**

**Myriam EL Khoury-Malhame**

CC: lchekuri@kansascity.edu, lchekuri@freemanhealth.com

PONE-D-24-53040R3

Cultural Stigma, Psychological Distress and Help-Seeking:

Moderating role of Self-esteem and Self-stigma

PLOS ONE

Dear Dr. El Khoury Malhame,

Thank you for submitting your manuscript to PLOS ONE. After careful consideration, we feel that it has merit but does not fully meet PLOS ONE’s publication criteria as it currently stands. Therefore, we invite you to submit a revised version of the manuscript that addresses the points raised during the review process.

Please submit your revised manuscript by Jul 24 2025 11:59PM. If you will need more time than this to complete your revisions, please reply to this message or contact the journal office at plosone@plos.org. When you're ready to submit your revision, log on to https://www.editorialmanager.com/pone/ and select the 'Submissions Needing Revision' folder to locate your manuscript file.

Peer Review Report

Manuscript Title: Cultural Stigma, Psychological Distress and Help-Seeking:

Moderating role of Self-esteem and Self-stigma in Lebanon

1. General Comments

The authors have responded to previous reviewer comments in this revision, and the

manuscript has improved in terms of data consistency and the alignment between

hypotheses and analyses. The study’s topic, concerning mental health stigma and its

correlates in the Lebanese cultural context, is of research value.

**Thank you, much appreciated**

However, for the manuscript to meet the standard for publication, several issues still

require attention. Revisions are recommended to further enhance the manuscript’s

scientific rigor, clarity, and accuracy in its interpretation, methodological reporting,

and language.

**This has been addressed accordingly.**

2. Specific Issues for Correction

2.1. Rigor in Interpretation and Language

Correction of Causal Language:The study employs a cross-sectional design, which

does not support causal conclusions. However, the text frequently uses language that

implies causality (e.g., “leading to” in the Abstract; “deters” and “exacerbating” in the

Discussion), which constitutes an over-interpretation of the findings. It is

recommended that the authors systematically revise the manuscript to replace such

terms with more precise, associative language (e.g., “is associated with,” “is related

to”) to ensure the conclusions are rigorously supported by the data.

**Absolutely on point comment. The team had previously edited the Introduction and has indeed shown less consistency in revisiting abstract and Discussion. A thorough review addressing the above has been done.**

2.2. Accuracy in Methods and Results Reporting

2.2.1 In the ‘Participants’ subsection of the Methods, the sentence “The number of

participant responses that were excluded from the analysis was 14 due to incomplete

survey responses.” is repeated verbatim. One instance should be removed.

**Done. Apologies for the shortsighted repetition.**

2.2.2 In the Results section, the sum of the percentages for different mental illness

diagnoses exceeds 100%. This suggests participant comorbidity, but this is not

explicitly stated. A brief explanatory note should be added to clarify this for the

reader.

**The explanation of comorbidity has been added accordingly. “Some participants reported comorbid diagnoses i.e., they documented being diagnosed with more than one mental disorder”**

2.3. Language and Formatting Corrections

2.3.1 Spelling Errors:

 In the ‘Focus on the Lebanese Context’ subsection, “loosers” should be corrected

to “losers”.

**Done.**

 In the ‘Limitations and Future Research’ subsection, “ofour” should be corrected

to “of our”.

**We couldn’t find this iteration**

2.3.2 Grammar and Punctuation:

 In the ‘Participants’ subsection, there is a stray space before the period in

“researchers .”.

**Done at the end of the procedure section, the period was in bold. The format has been corrected.**

 In the description of the Self-Stigma Questionnaire SSQ, the phrase “high level of

internal consistency, and Cronbach's alpha coefficients ranging from 0.75 to

0.901.” is a sentence fragment and should be corrected.

**Done. It now reads “The scale systematically shows a high level…”**

2.3.3 Phrasing and Word Choice:

 In the Discussion, “complexified” is non-standard and should be replaced with

“complicated”.

**Done**

 In the Statistical Analysis section, “adjusted over the following covariates” is

unidiomatic. The standard preposition is “for.”

**Done for the 2 instances**

 The phrase “The student t” is informal. It should be revised to the more standard

“A Student's t-test.”

**Absolutely. Apologies for the informality. This has been corrected**

3. Final Recommendation

The core research presented in the manuscript is sound. However, the manuscript

requires a thorough proofreading to address a number of basic errors before it can be

considered ready for publication. I strongly recommend that the authors conduct a

comprehensive review of the entire text to correct all errors in grammar, spelling,

punctuation, and capitalization, including the specific examples provided in this

report.

Once these essential corrections have been made, the manuscript will represent a

valuable contribution to the literature. Therefore, my recommendation is as follows:

Minor Revision.

**Amazing closeup attention to details and much needed recommendations. The team acknowledges the vast knowledge and the generosity of the reviewer with his/her time and energy.**
