# Supplementary material for: Cultural stigma, psychological distress and help-seeking: Moderating role of self-esteem and self-stigma in Lebanon
Source: PLoS One. 2025 Sep 25;20(9):e0315063. doi: 10.1371/journal.pone.0315063 (PMC12463265; doi:10.1371/journal.pone.0315063)
Supplement: Attachment — Submitted filename: Response to reviewers comments_230625.docx [file pone.0315063.s010.docx]

**Manuscript Title**: Cultural Stigma, Psychological Distress and Help-Seeking: Moderating role of Self-esteem and Self-stigma in Lebanon

**Dearest Editorial team**

**On behalf of my colleagues, we are grateful for the comments provided to help uplift our manuscript and meet journal criteria.**

**We are hereby providing a point-by-point reply to the editor and reviewers in blue color; and have highlighted in yellow tracked changes in the manuscript for ease of tracking. We have also conducted a thorough proofread of the manuscript.**

**Thank you for giving us the opportunity to give visibility to our work from the underrepresented part of the world.**

**To the Authors:**

Thank you for submitting the revised manuscript. The research topic is of significant interest and the study has the potential to make a valuable contribution to the field. To help the manuscript reach its full potential, this review focuses on issues of language and formatting that, if addressed, will greatly enhance the clarity and professionalism of the work. It is important to note that the manuscript still contains several basic errors that can be resolved with a thorough proofread. To ensure the scientific merit of the work is presented as clearly as possible, comprehensive proofreading by the authors is essential. This allows the peer review process to focus on the scientific content rather than on copy-editing. To that end, this review provides a detailed list of language and formatting issues that remain.

We strongly encourage the authors to not only correct these specific points but also to conduct a proactive and comprehensive review of the entire manuscript. A thoroughly proofread manuscript will allow reviewers to fully engage with the important research presented and will facilitate a smooth path toward final acceptance. **Done. Thank you for your encouragement and support.**

**Specific Issues for Correction**

**A. Spelling and Typographical Errors**

**1.Location: In the ‘Limitations and Future Research’ subsection.**

**Original Sentence**: “To the best ofour knowledge, this is the first study addressing stigma, self-stigma and self- esteem in a sample of young adults with existing mental disorders.”

**Problem**: ‘ofour’ is a typo and should be corrected to ‘of our’. **Thank you. This typo is now corrected in the manuscript.**

**2.Location: In the ‘Focus on the Lebanese Context’ subsection.**

**Original Sentence**: “Lebanon is a small Levantine country with a unique collectivist culture and a heterogenous landscape of 18 officially recognized religious groups (Faour, 2007).”

**Problem**: ‘heterogenous’ is a typo and should be corrected to ‘heterogeneous’. **Thank you. This typo is now corrected in the manuscript.**

**B. Grammar and Sentence Structure**

**3.Location: In the ‘BACKGROUND’ section**, under the ‘Stigma, Self-Stigma and Mental Distress subsection.

**Original Sentence**: “A significant body of research on mental health stigma and its deleterious impacts have for instance explained how the perception of individuals with schizophrenia as dangerous and commonly crazy have, among other, lead to internalized negative beliefs about oneself referred to as self-stigma (Yanos et al., 2015).”

**Problem**: The subject is “A significant body of research” (singular), so the verb must also be singular (has), not have. This is a subject-verb agreement error. **Thank you. The verb “have” is now replaced by “has” in the manuscript.**

**4.Location: In the ‘Measures’ subsection**, in the description of the Self-Stigma Questionnaire (SSQ).

**Original Sentence**: “The scale systematically shows high level of internal consistency, **and** Cronbach's alpha coefficients ranging from 0.75 to 0.901”

**Problem**: The part of the sentence after and is a sentence fragment; it lacks a main verb and breaks the parallel structure. “**and” has been replaced by “with” in the manuscript.**

**5.Location: In the ‘DISCUSSION section**.

**Original Sentence**: “The relationship between stigma, psychological distress and help-seeking intentions is further complicated and influenced by self-esteem as well as self- stigma**, that** are shown to play crucial mediating roles respectively.”

**Problem**: For this non-restrictive clause, ‘which’ should be used instead of ‘that’. The use of that after a comma is incorrect. **We have replaced “that” by “which” in the manuscript.**

**6.Location: In the ‘Conclusion section.**

**Original Sentence**: “Feedback, from focus group interviews post-workshop showed that the program succeeded in instilling favorable attitudes and beliefs about themselves...”

**Problem:** The comma after "Feedback" is unnecessary and incorrectly separates the subject from its verb, "showed," making the sentence structure awkward. **The comma after “Feedback” has been removed.**

**C. Wording and Clarity**

**7.**Location: In the ‘Procedure subsection.

**Original Sentence**: “The link was subsequently sponsored by targeting Lebanese 18-40 years users.” **Problem:** The phrasing is unidiomatic. It should be revised to the more standard “users aged 18-40” or “18- to 40-year-old users.” **We have now replaced “18-40 years users” by “****users aged 18-40”.**

**8.Location: In the ‘Statistical Analysis’ subsection.**

**Original Sentence**: “...since the skewness (= .001) and kurtosis (= -.702) values varied between -1 and +1 (Field, 2013).”

**Problem:** The word “varied” is used imprecisely here, as skewness and kurtosis are two fixed values. Using “were” or “fell within” would be more accurate. **The word “varied” has been replaced by “were”.**

**9.Location: In the ‘RESULTS’ section,** under the ‘Bivariate analysis of factors associated with attitude help seeking subsection.

**Original Sentence**: “The Bonferroni post-hoc analysis results showed that, in terms of age, there was a significance difference between the group aged 20-29 years and the following age groups...”

**Problem**: The adjective “significant” should be used here to modify the noun difference, not the noun “significance”. **We have replaced “significance” by the adjective “significant”. We apologize for this grammatical error.**

**D. Internal Consistency**

**10.Location: In the ‘Focus on the Lebanese Context’ subsection and the ‘References’ section**.

**Problem:** The text refers to the “National Mental Health Strategy for Lebanon” as covering the years (2023-2030), but the title of the strategy listed in the references section indicates the years are (2024- 2030). These dates are inconsistent and must be verified and unified. **We have verified the dates and replaced the year 2023 by 2024. We apologize for this typo error.**

**Final Recommendation**

Based on the detailed review above, my final recommendation is: Minor Revision. **Thank you**
