# Supplementary material for: Cultural stigma, psychological distress and help-seeking: Moderating role of self-esteem and self-stigma in Lebanon
Source: PLoS One. 2025 Sep 25;20(9):e0315063. doi: 10.1371/journal.pone.0315063 (PMC12463265; doi:10.1371/journal.pone.0315063)
Supplement: Attachment — Submitted filename: Peer Review Report 20250701.docx [file pone.0315063.s013.docx]

**Peer Review Report**

**Manuscript Title:** Cultural Stigma, Psychological Distress and Help-Seeking: Moderating Role of Self-Esteem and Self-Stigma in Lebanon

**Reviewer’s Comments:**

This manuscript has undergone six rounds of revision, and I appreciate the authors’ continued efforts to address reviewer feedback. However, several key issues—including terminological misusage, misalignment between analyses and interpretations, and inconsistent claims—still remain unresolved.

These are not minor oversights but recurring concerns that impact the clarity and validity of the study. To ensure real progress and avoid further delays, **I strongly encourage the authors to conduct a full, proactive review of the manuscript before resubmission. This includes carefully cross-checking all terminology, hypotheses, data interpretations, and summary statements across the entire text.**

I believe the manuscript has potential, but it now requires more than point-by-point responses—**it needs a self-directed, rigorous audit to reach publication standards.** I look forward to reviewing a substantially strengthened version, ideally with a more detailed and reflective response letter as well.

**Q1.**The authors use PROCESS Macro Model 1, which is clearly a moderation model:

“*The moderation analysis was conducted using PROCESS MACRO (an SPSS add-on), version 3.4, Model 1.*”(Methods section) Also:“*Self-esteem, stigma and self-stigma scores were included as moderators..*.” (Statistical Analysis)

**However, the abstract and discussion incorrectly refer to “mediating roles”**:“*...self-esteem as well as self-stigma, which are shown to play crucial mediating roles respectively.*” (Abstract and Discussion) This is a fundamental mischaracterization of the statistical method and must be corrected throughout the manuscript. Meanwhile, this sentence is not only incorrect in using “mediating,” but also misuses “respectively,” since there is no one-to-one mapping presented between moderators and specific paths.

**Q2.** Only self-esteem is included as a moderator in the hypotheses: “*H2: Self-esteem is hypothesized to moderate the relationship between psychological distress and help-seeking attitudes...*” (End of Introduction) **Yet self-stigma is also included in the moderation models later, without any prior theoretical framing or hypothesis.** This creates a disconnect between the theoretical model and statistical analysis.

***The authors are encouraged to incorporate a clear theoretical rationale for the moderating role of self-stigma earlier in the manuscript, ideally in the introduction, supported by relevant literature.*** Furthermore, all hypotheses would benefit from revision for greater academic clarity, specificity, and alignment with the final model.

If helpful, the authors may refer to hypothesis formulations in published studies on similar topics in *PLOS ONE* or related journals, particularly those addressing moderated relationships between psychological distress, self-stigma, self-esteem, and help-seeking (e.g., structured hypotheses that distinguish between direct, indirect, and moderated effects). This could enhance both conceptual clarity and methodological consistency.

**Q3.** Throughout the manuscript, the terms “stigma” and “self-stigma” are at times used interchangeably:“*Stigmatization, both general and internalized, is systematically associated with decreased help-seeking.*” (Abstract and Discussion) **However, these refer to different constructs, measured with distinct scales (KSS vs. SSQ)**. The manuscript should consistently differentiate external cultural stigma and internalized self-stigma to avoid conceptual confusion. The authors are encouraged to provide clear operational definitions of these two constructs in the introduction.

**Q4.** Table 2 shows that PhD (3.40) and high school (3.12) participants had higher help-seeking scores than bachelor (2.15) and master degree holders (2.37). **Yet the discussion incorrectly states**: “*University graduates hold considerably fewer negative attitudes toward therapy than both high school students and doctorate holders.*” (Discussion) The authors are encouraged to revise the discussion to accurately reflect the findings.

**Q5.** The discussion claims: “*Adults in their forties (40s) showed more positive attitudes towards help-seeking than younger adults.*” (Discussion) However, Table 1 shows only 2 participants in this age group: “40: 2 (.8%)” (Table 1) **Drawing any conclusion from such a tiny subsample is highly unreliable and should be clearly marked as non-generalizable.** The authors might consider qualifying the claim more cautiously.

**Q6. While the sample includes individuals from different education levels, the distribution is heavily skewed** (with 74.7% being bachelor’s degree holders). Therefore, repeated references to “various educational backgrounds” (e.g. “...from various educational backgrounds and located in different Lebanese regions.” “...including those from various educational backgrounds.”“...represented various educational backgrounds.”)may risk misleading readers regarding the sample’s representativeness and the generalizability of findings. The authors are encouraged to consider softening or more precisely qualifying such statements to better reflect the actual distribution of the sample. For example, expressions such as “predominantly bachelor-level backgrounds” or “mainly undergraduate-level participants with limited representation from other levels” would more accurately reflect the sample composition. It is also advisable to include this limitation in the relevant section.

**Q7.** Currently, the “Conclusion” section focuses solely on practice-based suggestions (e.g., engaging religious leaders, promoting drama therapy, leveraging social media), while entirely omitting a summary of empirical findings. Given that the manuscript already includes a separate **Discussion**/**Limitations and Future Research** section, this “Conclusion” does not function as such.

The authors are encouraged to restructure the final section with the following format:

**New Section:**

**Implications and Conclusions** (as a Level-1 heading), containing:

***Practical Implications*** (shortened from current content, max 2–3 paragraphs);

***Theoretical Implications*** (a new paragraph summarizing how findings contribute to stigma theory, help-seeking behavior models, and collectivist cultural contexts);

***Conclusions*** (a new paragraph summarizing the study’s main findings and contributions in approximately 150–200 words).

This structure will enhance coherence, align with academic conventions, and better communicate both empirical and applied value.
