# Supplementary material for: Cultural stigma, psychological distress and help-seeking: Moderating role of self-esteem and self-stigma in Lebanon
Source: PLoS One. 2025 Sep 25;20(9):e0315063. doi: 10.1371/journal.pone.0315063 (PMC12463265; doi:10.1371/journal.pone.0315063)
Supplement: Attachment — Submitted filename: Response to reviewers_140725.docx [file pone.0315063.s014.docx]

**Dearest editorial team**

**We have hereby provided point-by point comment in bold in blue and have highlighted the tracked changes in the manuscript accordingly.**

**Peer Review Report**

**Manuscript Title:** Cultural Stigma, Psychological Distress and Help-Seeking: Moderating Role of Self-Esteem and Self-Stigma in Lebanon

**Reviewer’s Comments:**

This manuscript has undergone six rounds of revision, and I appreciate the authors’ continued efforts to address reviewer feedback. However, several key issues—including terminological misusage, misalignment between analyses and interpretations, and inconsistent claims—still remain unresolved.

These are not minor oversights but recurring concerns that impact the clarity and validity of the study. To ensure real progress and avoid further delays, I strongly encourage the authors to conduct a full, proactive review of the manuscript before resubmission. This includes carefully cross-checking all terminology, hypotheses, data interpretations, and summary statements across the entire text. I believe the manuscript has potential, but it now requires more than point-by-point responses—it needs a self-directed, rigorous audit to reach publication standards. I look forward to reviewing a substantially strengthened version, ideally with a more detailed and reflective response letter as well.

**We deeply appreciate your thorough and continued engagement with our work, by now, reviewers are practically co-authors in spirit.**

**While diligently working to address each point raised across the previous rounds, a bird’s-eye, integrative review was indeed warranted to smooth sentences and findings.**

**We have conducted a full-scale internal audit of the manuscript as per the suggestion and have:**

- **focused on terminological precision,**
- **aligned analyses and interpretations,**
- **capitalized on overall conceptual clarity.**

**Our revised submission reflects this broader self-directed effort to strengthen coherence and academic rigor. Thank you for your patience, persistence, and for nudging us from “good intentions” to “publication-ready.”**

**Q1.** The authors use PROCESS Macro Model 1, which is clearly a moderation model:

“*The moderation analysis was conducted using PROCESS MACRO (an SPSS add-on), version 3.4, Model 1.*”(Methods section) Also: “*Self-esteem, stigma and self-stigma scores were included as moderators..*.” (Statistical Analysis)

However, the abstract and discussion incorrectly refer to “mediating roles”:“*...self-esteem as well as self-stigma, which are shown to play crucial mediating roles respectively.*” (Abstract and Discussion) This is a fundamental mischaracterization of the statistical method and must be corrected throughout the manuscript. Meanwhile, this sentence is not only incorrect in using “mediating,” but also misuses “respectively,” since there is no one-to-one mapping presented between moderators and specific paths.

**We have corrected this typo mistake and sentence fragment and have revised the abstract and manuscript for clarity. Our study indeed focuses on moderating roles and not mediation investigation. The factors of self-esteem and self-stigma have been investigated separately and not “respectively”. Thank you for the comment.**

Q2. Only self-esteem is included as a moderator in the hypotheses: “*H2: Self-esteem is hypothesized to moderate the relationship between psychological distress and help-seeking attitudes...*” (End of Introduction) Yet self-stigma is also included in the moderation models later, without any prior theoretical framing or hypothesis. This creates a disconnect between the theoretical model and statistical analysis.

*The authors are encouraged to incorporate a clear theoretical rationale for the moderating role of self-stigma earlier in the manuscript, ideally in the introduction, supported by relevant literature.* Furthermore, all hypotheses would benefit from revision for greater academic clarity, specificity, and alignment with the final model.

If helpful, the authors may refer to hypothesis formulations in published studies on similar topics in *PLOS ONE* or related journals, particularly those addressing moderated relationships between psychological distress, self-stigma, self-esteem, and help-seeking (e.g., structured hypotheses that distinguish between direct, indirect, and moderated effects). This could enhance both conceptual clarity and methodological consistency.

**The hypotheses have been edited to align with the moderation terminology used in the PROCESS analysis framework.**

**H1: Self-esteem will moderate the relationship between psychological distress and help-seeking attitudes, such that the negative association between psychological distress and help-seeking will be weaker at higher levels of self-esteem.**

**H2: Self-stigma will moderate the relationship between psychological distress and help-seeking attitudes, such that individuals with higher self-stigma may show less favorable help-seeking attitudes when experiencing psychological distress.**

Q3. Throughout the manuscript, the terms “stigma” and “self-stigma” are at times used interchangeably: “*Stigmatization, both general and internalized, is systematically associated with decreased help-seeking.*” (Abstract and Discussion) However, these refer to different constructs, measured with distinct scales (KSS vs. SSQ). The manuscript should consistently differentiate external cultural stigma and internalized self-stigma to avoid conceptual confusion. The authors are encouraged to provide clear operational definitions of these two constructs in the introduction.

**The intent of the study is to indeed introduce the 2 different concepts of stigma and self-stigma. The operational definition has been highlighted in the introduction in the segment on stigma, self-stigma, and mental distress, and further revisited throughout the entire manuscript.**

**Yet, at times, the authors point out that both work in the same direction, as was set in the abstract in relation for example with diminished help-seeking attitudes. This has been changed to “Stigmatization, in both forms i.e., external/cultural, and internalized,” to avoid confusion.**

Q4. Table 2 shows that PhD (3.40) and high school (3.12) participants had higher help-seeking scores than bachelor (2.15) and master degree holders (2.37). Yet the discussion incorrectly states: “*University graduates hold considerably fewer negative attitudes toward therapy than both high school students and doctorate holders.*” (Discussion) The authors are encouraged to revise the discussion to accurately reflect the findings.

**Thank you for pointing out this. After revising the discussion section, we have removed this paragraph to avoid confusing the reader as the bivariate analysis is not the main focus of this study.**

Q5. The discussion claims: “*Adults in their forties (40s) showed more positive attitudes towards help-seeking than younger adults.*” (Discussion) However, Table 1 shows only 2 participants in this age group: “40: 2 (.8%)” (Table 1) Drawing any conclusion from such a tiny subsample is highly unreliable and should be clearly marked as non-generalizable. The authors might consider qualifying the claim more cautiously.

**We removed this paragraph since it discusses results from the bivariate analysis, which is not the main focus of this study.**

Q6. While the sample includes individuals from different education levels, the distribution is heavily skewed (with 74.7% being bachelor’s degree holders). Therefore, repeated references to “various educational backgrounds” (e.g. “...from various educational backgrounds and located in different Lebanese regions.” “...including those from various educational backgrounds.”“...represented various educational backgrounds.”)may risk misleading readers regarding the sample’s representativeness and the generalizability of findings. The authors are encouraged to consider softening or more precisely qualifying such statements to better reflect the actual distribution of the sample. For example, expressions such as “predominantly bachelor-level backgrounds” or “mainly undergraduate-level participants with limited representation from other levels” would more accurately reflect the sample composition. It is also advisable to include this limitation in the relevant section.

**Thank you for your thoughtful comment. We have replaced “various educational backgrounds” in the abstract and Participants’ section by “predominantly bachelor-level backgrounds” and added the following sentence in the limitation section.**

***Our study indeed focused on educated young adults and should thus be generalized with care. Future studies should consider involving individuals with varying levels of education and broader age ranges.***

Q7. Currently, the “Conclusion” section focuses solely on practice-based suggestions (e.g., engaging religious leaders, promoting drama therapy, leveraging social media), while entirely omitting a summary of empirical findings. Given that the manuscript already includes a separate Discussion/Limitations and Future Research section, this “Conclusion” does not function as such.

The authors are encouraged to restructure the final section with the following format:

New Section:

**Implications and Conclusions** (as a Level-1 heading), containing:

***Practical Implications*** (shortened from current content, max 2–3 paragraphs);

***Theoretical Implications*** (a new paragraph summarizing how findings contribute to stigma theory, help-seeking behavior models, and collectivist cultural contexts);

***Conclusions*** (a new paragraph summarizing the study’s main findings and contributions in approximately 150–200 words).

This structure will enhance coherence, align with academic conventions, and better communicate both empirical and applied value.

**This is a very helpful recommendation. The authors have actioned it accordingly. The practical implications reflect steps within the community whereas the theoretical implications call to upgrade the mentioned stigma theories. Lastly the conclusion sums up ways forward in local, regional, and global context.**

***Practical Implications***

***This study sheds light on the significance of mental health stigma on psychological distress and help-seeking. Findings highlight the pivotal role of both personal and social targets in addressing the matter, especially given that self-esteem and self-stigma were found to moderate the above intricate relationships. In a collectivist Lebanese context, this means capitalizing on social connectedness by intensifying early awareness within appropriate cultural norms.***

***At a local level, contact with community leaders, known as Mukhtars, and religious figures to address stigma, is essential to bridge the gap between the Non-Governmental Organizations (NGOs) mental health workers and communities. Those can be seen as outsiders who come to change the traditional norms, especially in a sectarian country such as Lebanon (Osman & Chebaro, 2024). To this extent, culturally sensitive interventions that promote mental health literacy, and challenge misconceptions should be advocated along local leaders including religious clergy; whom have the trust of the community and have a crucial role in referring those who might initially seek help from religious figures or attribute mental health issues to spiritual factors (Osman & Chebaro, 2024).***

***Other actionable steps would include actively fostering youngsters’ self-esteem within families, societies, schools, universities, and municipalities with specific evidence-based programs aimed at improving self-esteem and reducing stigma. One such program significantly enhanced emotional intelligence skills in public high-school students, while also targeting self-esteem and self-stigma (Sanchez-Ruiz et al., 2023). Focus group interviews conducted after the workshop showed that the program effectively instilled favorable self-attitudes and beliefs about addressing psychological distress. In particular, the program helped participants manage and overcome feelings of inferiority and shame concerning mental disorders and individuals experiencing them.***

***Another practical measure involves using social media platforms such as WhatsApp and Instagram to facilitate open dialogue about mental health and build public empathy. This approach has been supported by studies in stigmatized collectivists nations such as Lebanon (Osman & Chebaro, 2024) and Malaysia (Rahman et al., 2022). To this extent, people who might have depression or experience anxiety can connect to other Lebanese people, and to mental health specialists and in turn override feelings of loneliness, isolation, and helplessness.***

***Theoretical Implications***

***The present study provides important theoretical insights into the interplay between stigma, help-seeking behavior, and psychological distress within a collectivist cultural context. Our findings substantiate and extend Stigma Theory by providing evidence that both public and internalized stigma are not only prevalent but deeply interwoven with self-perceptions and psychological suffering in such contexts. The moderating roles of self-esteem and self-stigma confirm key tenets of Stigma Management Theory, particularly the idea that personal resources and internalized beliefs shape individuals’ coping strategies in the face of societal rejection.***

***Finally, in collectivist cultures like Lebanon, where family honor and communal belonging heavily influence identity, our findings suggest that inasmuch as social values offer community support (Osman & Chebaro, 2024), they can negatively influence help-seeking as stigma carries a heightened threat in some social circles. To avoid worsening such internal conflicts, this study urges culturally adapted theories of mental health behavior that account for communal interdependence and honor-based identity systems. Promising success of interventions building on comedy and group cohesion such as drama therapy have also shown efficacy and cultural sensitivity in coming together to heal and overcome stigma (Haddad et al., 2024). These would be particularly relevant as they would factor in cultural resources such as the reported collective sense of humor, used to laugh at shared miseries and to cope with the country’s latest socio- economic and political tensions.***

***Conclusion***

***This study contributes to the limited but growing literature on mental health stigma in the Arab world by examining how stigma, psychological distress, and individual-level moderators such as self-esteem and self-stigma shape help-seeking attitudes in a Lebanese sample. Findings could be relevant in the Global south faced with protracted crises and in the Global North with incrementally rising rates of mental distress. We build on recommendations from the Lancet Commission on ending stigma and discrimination in mental health (2022), suggesting nuanced internal processes may either buffer or compound the effects of stigma. Since approaching people with direct lived experiences of psychological disorders, our research could inform policy makers in comparable Levantine cultures to increase help-seeking attitudes by capitalizing on positive mental health literacy to build self-esteem and contribute to individual-resilience, while incentivizing proactive efforts to collectively destigmatize and treat mental illness. This would lead to favorable outcomes for both mental and physical health of vulnerable young adults.***

**Best regards,**

**Corresponding author**
