# Supplementary material for: Cultural stigma, psychological distress and help-seeking: Moderating role of self-esteem and self-stigma in Lebanon
Source: PLoS One. 2025 Sep 25;20(9):e0315063. doi: 10.1371/journal.pone.0315063 (PMC12463265; doi:10.1371/journal.pone.0315063)
Supplement: Attachment — Submitted filename: Stigma_Review_120825.docx [file pone.0315063.s015.docx]

Reviewer #1: Thank you for your patience and effort. After seven rounds of detailed revisions, I am pleased to see that the manuscript's overall quality, logical rigor, and clarity of argument have all improved very significantly. Based on these important improvements, I will be recommending acceptance to the editorial office.
**We are even more thankful for the patience and the grit in making this publication happen. A Million thanks**

Before the manuscript proceeds to the next stage, I would like to suggest two final, minor points for polishing to ensure the work is presented as professionally as possible:

1. In the discussion on the moderating role of self-stigma, a sentence reads: "Within this framework, we hypothesize that self-stigma may serve as a protective factor...". This statement is quite confusing as it appears to directly contradict your findings. According to your data in Table 5, high levels of self-stigma actually function as a risk factor, exacerbating the negative relationship between psychological distress and help-seeking attitudes. I would recommend thoroughly revising this sentence to ensure it aligns with your data.
**We rephrased the idea as suggested. Thank you.**

2. The manuscript uses a numerical citation system (e.g., [68]), but in some places, this is combined with an author-year format, such as in "...when subjected to stigma [68] (El Hayek et al., 2021).". This is redundant. I suggest a final check of the manuscript to remove such redundancies, leaving only the numerical citations for consistency and adherence to the journal's style.

**Corrected.**

**We would like to thank all the editorial team again and again for all your efforts and time.**

**This manuscript has allowed us to grow in ways we never imagined <3**
